# Unveiling Cryptic Species Diversity and Genetic Variation of *Lasiodiplodia* (Botryosphaeriaceae, Botryosphaeriales) Infecting Fruit Crops in Taiwan

**DOI:** 10.3390/jof9090950

**Published:** 2023-09-20

**Authors:** Ya-Zhu Ko, Wasantha Kumara Liyanage, Huei-Chuan Shih, Min-Nan Tseng, Meng-Shin Shiao, Yu-Chung Chiang

**Affiliations:** 1 Department of Biological Sciences, National Sun Yat-sen University, Kaohsiung 804, Taiwan; abscl77512@gmail.com; 2 Department of Agricultural Biology, Faculty of Agriculture, University of Ruhuna, Kamburupitiya 81100, Sri Lanka; wasanthk2011@gmail.com; 3 Department of Nursing, Meiho University, Pingtung 912, Taiwan; x00002213@meiho.edu.tw; 4 Kaohsiung District Agricultural Research and Extension Station, Ministry of Agriculture, Pingtung 908, Taiwan; 5 Research Center, Faculty of Medicine Ramathibodi Hospital, Mahidol University, Bangkok 73170, Thailand; msshiao@gmail.com; 6 Department of Biomedical Science and Environment Biology, Kaohsiung Medical University, Kaohsiung 807, Taiwan; 7 The Multidisciplinary and Data Science Research Center (MDSRC), National Sun Yat-sen University, Kaohsiung 804, Taiwan

**Keywords:** *Lasiodiplodia*, phylogeny, genetic diversity, host-associated differentiation

## Abstract

The genus *Lasiodiplodia*, a member of the family Botryosphaeriaceae, is an important fungal disease genus in agriculture. However, the *Lasiodiplodia* species survey and genetic diversity in Taiwan remain unclear. This study aimed to investigate the *Lasiodiplodia* species associated with various fruit species to explore the cryptic *Lasiodiplodia* species diversity, validate species delimitation, and unveil cryptic genetic diversity. Overall, six *Lasiodiplodia* species were identified, with several new records of infection identified. Additionally, phylogenetic analyses indicated that the relations of all isolates of *L. theobromae* might be paraphyletic. They were grouped with *L. brasiliense* based on Automatic Barcode Gap Discovery (ABGD), Automatic Partitioning (ASAP) and structure-based clustering analyses. These analyses did not provide conclusive evidence for *L. brasiliensis* as a stable species. It may be necessary to gather more information to clarify the species delineation. The multiple new records of *Lasiodiplodia* species with high genetic diversity and differentiation revealed that the diversity of *Lasiodiplodia* in Taiwan was underestimated in the past. We found that *L. theobromae* has the highest number of haplotypes but the lowest number of haplotype and nucleotide diversities, indicating a recent population expansion. This was supported by the significant negative Tajima’s *D* and Fu and Li’s D* tests. The high genetic diversity, low gene flow, and host-associated differentiation of *Lasiodiplodia* species indicate that they might harbour powerful evolutionary potential in Taiwan. This study provided critical insights into genetic variation, host-associated differentiation, and demography of *Lasiodiplodia* species, which would be helpful for disease management of related pathogens.

## 1. Introduction

The genus *Lasiodiplodia* belongs to the family Botryosphaeriaceae, with more than 80 species reported in the MycoBank database (https://www.mycobank.org/, accessed on 18 June 2023). The genus was widely studied due to its pathogenic nature to many important agricultural plants in tropical and subtropical areas, with thousands of plant hosts recorded [1,2]. Out of all the host plants that have been documented, mango, cocoa, and grape have the most comprehensive records of coexisting infections with various *Lasiodiplodia* species [3,4,5,6]. More than 70 *Lasiodiplodia* species have been reported and described since 2004 based on morphological characteristics and molecular data. However, many *Lasiodiplodia* species have overlapping morphological characteristics [7,8] or lack morphological character descriptions, such as *L. endophytica* [9]. Furthermore, out of all legitimate *Lasiodiplodia* species, only five of them have sexual morphs described [8,10,11,12,13].

Accurate and prompt diagnosis of plant fungal pathogens is crucial for disease management [14]. However, correct identification of the species in the genus *Lasiodiplodia* remains difficult for several reasons. First, identifying species based on morphological characteristics alone is unreliable, as the characteristics may vary within a species at different developmental stages or may be influenced by environmental factors [9,15]. Second, even if the species can be identified using the spore characteristics, obtaining single spore colonies is time consuming and requires specialized techniques, which are not easily applied in the field. Third, the symptoms caused by different *Lasiodiplodia* species are mostly indistinguishable, which include rots in various organs of the host plants (seeds, fruit, stem-end, panicle, neck), necrotic lesions, foliage yellowing; decline, branch dieback, gummosis and stem canker [3,6,7,16,17,18,19,20,21,22,23]. Additionally, these symptoms may be mistaken for those of other Botryosphaeriaceae species diseases [6,20,24,25,26,27]. Lastly, the coexisting infections by different species in this genus are commonly seen and may have a synergistic effect, which increases the complexities in disease management [3,4,5,6,17].

Molecular markers provide advantages in studying species diversity and evolution and contribute significantly to accurately identifying intricate fungal species [28,29]. The massive increase in molecular marker sequences and related phylogenetic analysis has dramatically influenced the systematics and taxonomy of many important plant pathogenic fungi, such as the genus *Lasiodiplodia* [24,30,31,32,33]. Several molecular markers have been used in identifying species in the genus *Lasiodiplodia*, including the internal transcribed spacer (ITS) of genomic rDNA, the nuclear ribosomal small subunit ribosomal RNA (SSU) gene, nuclear ribosomal large subunit ribosomal RNA (LSU) gene, translation elongation factor 1-alpha (TEF1), β-tubulin 2 gene (TUB2) and the mitochondrial ribosomal small subunit ribosomal RNA (mtSSU) gene [8,24,26]. The combination of using ITS and EF1 sequences provided sufficient resolution in identification at the species level in many genera of Botryosphaeriaceae [33]. However, it is insufficient for a robust phylogenetic identification for *Diplodia*, *Neofusicoccum*, and *Lasiodiplodia*. Additional loci, i.e., RPB2 and TUB2, must be considered for more robust analysis [33]. It is essential to use at least four loci to determine the uniqueness of each species to avoid questionable or unreliable identification of closely related species in the genus *Lasiodiplodia* [30].

Studies of *Lasiodiplodia* in Taiwan have been reported in several agricultural hosts, including guava [34], mango [35], papaya [36], avocado [37], jackfruit [38], kumquat [39], *Aquilaria sinensis* [40], Araucariaceae [41], lima bean [42], Malabar chestnut [43], and *Passiflora edulis* [41]. It is crucial to highlight that previous identifications of *Lasiodiplodia* in Taiwan were primarily based on morphological characteristics, and molecular information was very limited. As a result, the phylogenetic diversity of *Lasiodiplodia* in Taiwan is likely underestimated. A better understanding of pathogenic fungi will provide more insights into future disease management and prevention in agricultural fruit plants. Thus, this study aimed to investigate cryptic species of the genus *Lasiodiplodia* and their genetic diversity in Taiwan by (i) investigating *Lasiodiplodia* species in infected host plants to understand the prevalence and pathogen distribution; (ii) identifying the phylogenetic relations and species diversity of *Lasiodiplodia* species and its close related genus using a panel of molecular markers, i.e., combined SSU, ITS, TEF1, and TUB2; and (iii) evaluating the diversity, recombination, and demographics of the species.

## 2. Materials and Methods

### 2.1. Sample Collection and Single Spore Colony Isolation

The collected samples were surface sterilized with 70% ethanol for 2 min. Small samples were then taken from the interface between healthy and diseased tissue using a sterile scalpel and washed twice with sterile distilled water. The cut sample block was placed on potato dextrose agar (PDA, Difco) medium and incubated at 25 °C for seven days. After the cultured diseased tissue grew hyphae, the single hyphae were cut out at the colony’s edge and transferred to fresh media. The isolates were cultured in 2% water agar (WA) covered with sterilized sprigs of *Casuarina equisetifolia* on the surface as a substrate at 25 °C for four weeks to induce pycnidia formation. After sporulation, a single spore culture was obtained and transferred to fresh PDA plates. Morphological characteristics, such as the isolate’s shape, size, and colour, were observed under the microscope to assist in identifying the species of isolates. The conidia length and width were measured from different isolates, and 20 measurements were performed for each isolate to calculate the average, standard deviation, and 95% confidence interval.

Each fungal species’ isolation frequency (IF; %) was calculated as the number of collected tissues infected by each fungal confirmed by molecular identification divided by the total number of collected tissues incubated. A one-way ANOVA statistical analysis was performed using the aov() function in R version 4.3.1 software [44]. When F values were significant (*p* < 0.05), a post hoc Tukey test function TukeyHSD() from the {stats} package was used. A *p*-value was considered significant when lower than 0.01 to evaluate significant differences (*p* < 0.001) in isolation frequency among different Botryosphaeriaceae species for each host plant separately. Figures were created using the ggplot2 package in R version 4.3.0 software to draw a plot with error bars [45].

### 2.2. DNA Extraction and Sequencing of Four Markers

The mycelium was grown on PDA cultured at 25 °C and harvested for four weeks. A sterile scalpel was used to scrape mycelium to obtain a sample of 0.5 g. Then stainless steel beads, glass sand and 80 µL of 0.5 N NaOH were added to grind the samples at 4.0 m/s for 15 s using the FastPrep^®^-24 homogenizer (MP Biomedicals, Illkirch, France). The process was repeated twice to ensure that samples were homogenized entirely. The genomic DNA samples were extracted using the Genomic DNA Extraction Kit (RBC Bioscience, Taipei, Taiwan) from the paste-like mycelium preparations aforementioned. All extracted genomic DNA quality was examined using 1% agarose gel electrophoresis and the Lambda marker (200 µg/mL, Promega, Madison, WI, USA) as the standard to compare the extracted DNA concentration. Finally, the sterile water was used to adjust the extracted DNA concentration to 20 ng/µL for subsequent experiments and stored at −80 °C until further analysis.

A 50 µL PCR mix containing 1.5 µL of template DNA, 5 µL of 10 × reaction buffer, 5 µL of dNTP Mix (2 mM), 5 µL of each forward and reverse primers (2 mM), 1 µL of Taq polymerase (0.2 U µL-1; Promega), and 27.5 µL of sterile water was used for PCR. The ITS, TEF1, SSU and TUB2 regions were amplified with primer pairs ITS1 (5′-TCCGTAGGTGAACCTGCGG-3′) and ITS4 (5′-TCCTCCGCTTATTGATATGC-3′) [46], EF1-728F (5′-CATCGAGAAGTTCGAGAAGG-3′) and EF1-986R (5′-TACTTGAAGGAACCCTTACC-3′) [47], NS1 (5′-GTAGTCATATGCTTGTCTC-3′) and NS4 (5′-CTTCCGTCAATTCCTTTAAG-3′) [46], and Bt2a (5′-GGTAACCAAATCGGTGCTGCTTTC-3′) and Bt2b (5′-ACCCTCAGTGTAGTGACCCTTGGC-3′) [48]. PCR amplification reaction conditions: 95 °C for 5 min, followed by 35 cycles of 1 min at 94 °C, 1 min at 50–54 °C, 1 min at 72 °C, and the final extension of 10 min at 72 °C. All PCR fragments were purified with the HiYield™ Gel/PCR DNA Fragments Extraction Kit (RBC Bioscience). Both strands of the PCR fragments were sequenced using the ABI PRISM 3730XL DNA sequencer (Applied Biosystems, Foster City, CA, USA). The GenBank accession numbers are as follows: OR534007-OR534219 for ITS region; OR552184–OR552396 for TEF1 region, OR534310-OR534522 for SSU region and OR551773–OR551985 for TUB2.

### 2.3. Phylogenetic Analysis and Cryptic Species Analyses

The forward and reverse primer sequence data obtained from sequencing were aligned into contigs using the SeqMan software from the DNASTAR package (DNASTAR Inc., Madison, WI, USA). The alignments were edited and adjusted manually as needed. The resulting consensus sequence data were compared with the National Center for Biotechnology Information (NCBI) database using the BLASTN algorithm (http://www.ncbi.nlm.nih.gov/BLAST/, accessed on 18 June 2023)), with a query cover of 99–100% and an identity of 98–100% for species identification. Multiple sequences were aligned and retrieved from the GenBank dataset using MEGA 11 [49] for subsequent phylogenetic analyses.

The four phylogenetic analysis methods (Neighbor-Joining, Maximum Parsimony, Maximum Likelihood, and Bayesian inference) were used to perform phylogenetic classification based on multilocus sequence data. The best-fit nucleotide substitution model was determined using jModelTest v 2.1.7 [50] with the Akaike information criterion (AIC). The model was found to be HKY + G and was used in the Maximum Likelihood (ML) and Bayesian Inference (BI) analyses.

The Neighbor-Joining tree (NJ) was constructed using MEGA 11 [49] with the Kimura-two-parameter nucleotide substitution model for estimating genetic differences [51]. The bootstrap value was calculated based on 5000 resamplings. The NJ trees were generated for each locus alignment to evaluate the species resolution ability of loci.

The consensus Maximum Parsimony tree (MP) was inferred using the program SEQBOOT, DNAPARS, and CONSENSUS from PHYLIP v.3.698 [52]. A 1000 bootstrap replicates were performed with the SEQBOOT. DNAPARS produced the multiple Maximum Parsimony trees using a heuristic algorithm. CONSENSUS summarized to receive a consensus tree with the majority rule method.

The Maximum Likelihood (ML) analysis was carried out using RAxML v.8.2.10 [53], with a standard non-parametric bootstrap evaluation under the HKY + G nucleotide substitution model of evolution. The robustness of the analysis was evaluated by bootstrap support (MLBS) using the number of replicates automatically. The non-parametric bootstrap validation was set to 10 runs and 1000 replicates. Nodes with bootstrap values ≥ 50 were considered supported.

The Bayesian Inference (BI) analysis was conducted by MrBayes 3.2.7a [54]. With two independent runs of four chains for 40 million generations and sampling tree topologies every 100,000 generations. These values had accepted after 25% of the generations were discarded from each run as the burn-in phase. Convergence was ascertained by examining the average standard deviation of split frequencies (ASDSF) as well as the potential scale reduction factor (PSRF). PSRF values were accepted as close to 1.0 for all parameter estimates. ASDSF values dropping below 0.01 is a good convergence indication [55]. The tree topologies information of branch rates, node heights, and other statistics were visualized in Figtree v.1.4.4 [56].

To further validate species delimitation and unveil the cryptic species diversity of *Lasiodiplodia*, this study employed two DNA-based species delimitation methods: Automatic Barcode Gap Discovery (ABGD) and Automatic Partitioning (ASAP), as described by Puillandre et al. (2012) and (2020) [57,58], respectively.

ABGD approach sorts the multiple alignment sequences into hypothetical species based on the detection of breaks in the distribution of pairwise genetic distances (barcode gaps), which can be observed when the nucleotide divergence among the isolates belonging to the same species is smaller than the nucleotide divergence among the isolates from different species. Barcode gaps were detected and used to partition the data. Gaps detection and limit were inferred and then recursively applied to prior obtained groups to obtain the maximum number of groups [57]. This study used the combined four loci dataset, with the default setting, and genetic distances were calculated using the *p* distance model with minimum gap width set X = 1.5.

On the other hand, the ASAP method utilizes a hierarchical clustering algorithm that does not require prior knowledge of distance thresholds or phylogeny reconstruction [58]. Instead, it computes the *p*-value and relative barcode gap width and combines them into an ASAP score that ranks the partitions. The study utilized the *p*-distance model with default settings for the combined dataset.

### 2.4. Assessment of Nucleotide Diversity, Recombination, Linkage Disequilibrium and Demographic History

The genetic diversity and polymorphism were estimated using DnaSP v.6.12.03 [59]. Genetic diversity among *Lasiodiplodia* species was estimated by computing the number of haplotypes (*h*), segregating sites (*S*), haplotype diversity (*Hd*), and nucleotide diversity (*π*) [60]. Haplotype diversity (gene diversity) means the probability that two stochastically sampled alleles are different. Nucleotide diversity means the average number of nucleotide differences per site between randomly pairwise comparisons of DNA sequences [60].

The minimum number of recombination events (*R_M_*) values in the history for the *Lasiodiplodia* samples was further obtained using the four-gamete test [61] by DnaSP v.6.12.03. The linkage disequilibrium for the whole data was calculated by DnaSP v.6.12.03. Kelly’s *Z_nS_* statistic [62] for linkage disequilibrium is the average of *r*^2^ of the allelic identity over all pairwise comparisons between two loci [63]. The range of *Z_nS_* values is from 0 to 1, with higher values denoting stronger linkage disequilibrium [64]. Rozas’ *ZZ* [65] is the comparison between *Z_nS_* and *Z_A_* (*ZZ* = *Z_A_* − *Z_nS_*), where *Z_A_* is the average of *r*^2^ of the allelic identity between adjacent polymorphic sites overall pairwise comparisons.

To assess the past population expansion, two statistical tests were performed for each gene using the DnaSP v.6.12.03 software [59]: Tajima’s *D* test [66] and Fu and Li’s D* test [67]. Demographic and spatial expansion null hypotheses were tested by mismatch distribution analysis using 1000 parametric bootstraps [68], and the sum of squared deviations (SSD) and the Harpending’s raggedness index (*H*_Rag_) were applied to appraise the goodness-of-fit for each expansion mode [69]. In addition, to test for demographic processes, we used a pairwise mismatch distribution with the DnaSP v.6.12.03 software [59]. Mismatch curves were created for each *Lasiodiplodia* species and compared with the expected curves for the constant population size model and the population growth–decline model. An unimodal distribution indicates sudden population expansion [70], while a multimodal mismatch distribution indicates recent demographic expansion following population size reduction due to population subdivision [71].

### 2.5. Genetic Differentiation and Genetic Structure Analysis

The hierarchical analysis of molecular variance (AMOVA) via the Arlequin v.3.5 programs [72] was employed to examine variance partitioning into components derived from different levels and to assess the *F*-statistics significance using 999 permutations. The *F*-statistics can assess the degree of genetic differentiation [73]. *F*-statistics provide a basis for studying gene flow by comparing allele frequencies of polymorphic loci to detect subdivisions [74]. The *F_ST_* value can reflect the degree of genetic differentiation among populations. *F_ST_* = 1/(1 + 4 *Nm* for diploids; 1 + 2 *Nm* for haploids) [75], N is the effective population size, and m is the migration rate between populations. *Nm* is the haploid number of migrants coming into the population per generation.

The model-based clustering method in STRUCTURE v.2.3.4 [76] is used to calculate allele frequencies and assign related individuals to source populations based on likelihood. STRUCTURE applies a Bayesian iterative algorithm using Markov Chain Monte Carlo (MCMC) estimation to analyze differences in genetic variation distribution among defined populations and group individuals with similar variations. The MCMC process randomly assigns individuals to a predetermined number of K genetically ancestry clusters, estimates the variant frequency in each cluster, and reassigns individuals. The posterior probabilities of K clusters are calculated for each individual using 10^5^ burn-in periods followed by 10^6^ Monte Carlo Markov Chain (MCMC) replicates collected after burn-in to receive accurate parameter estimates. Twenty independent runs of the Markov chain were performed to verify the convergence of the chain and the consistency across each prior K run and ΔK was used to evaluate the actual K value [77]. The output file of STRUCTURE analysis was integrated and evaluated using the STRUCTURE HARVESTER program to visualize and evaluate the likelihood values across multiple K values and multiple iterations [78]. The most suitable K value was selected where CLUMPP aligns the multiple replicates of the choice K cluster in the STRUCTURE analysis results to help solve the multimodality problems [79]. The POPHELPER v.2.3.1 R package creates bar plots to present STRUCTURE output [80].

## 3. Results

### 3.1. Species Identification from Single Spore Colonies

**Figure 1:**
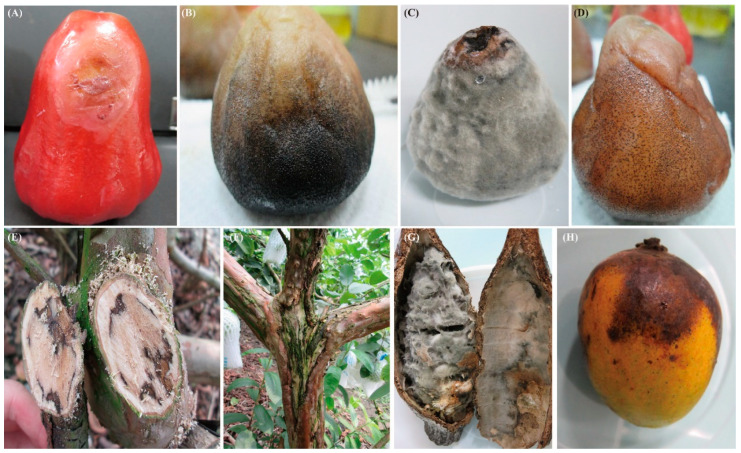
Disease symptoms of fruit rot and stem canker associated with *Botryosphaeriaceae* infection on different host species. (**A**) Fruit rot symptom on wax apple infected with *L. brasiliensis*. (**B**,**C**) Fruit rot symptom on wax apple infected with *L. theobromae*. (**D**) Fruit rot symptom on wax apple infected with *Neofusicoccum parvum*. (**E**) Canker symptom and internal tissue necrosis on the stem of guava infected with *L. theobromae*. (**F**) Linear canker symptom on the stem of guava infected with *L. theobromae*. (**G**) *L. theobromae* causes the charcoal pod rot of cocoa. (**H**) The symptom of fruit rot on mango infected with *L. theobromae*.

We collected infected stems from *Mangifera indica* (mango), *Psidium guajava* (guava), and *Cordia dichotoma*. Infected fruit samples were taken from mango, *Syzygium samarangense* (wax apple), *Carica papaya* (papaya), *Theobroma cacao* (cocoa), *Annona squamosa* (sugar apple), *Musa basjoo* (banana), and *Syzygium taiwanicum*, and infected flowers were taken from *Alpinia* (Figure 1, Table 1 and Table S1). The infected parts exhibited symptoms such as fruit rot, necrotic lesions, or neck rot.

**Figure 2:**
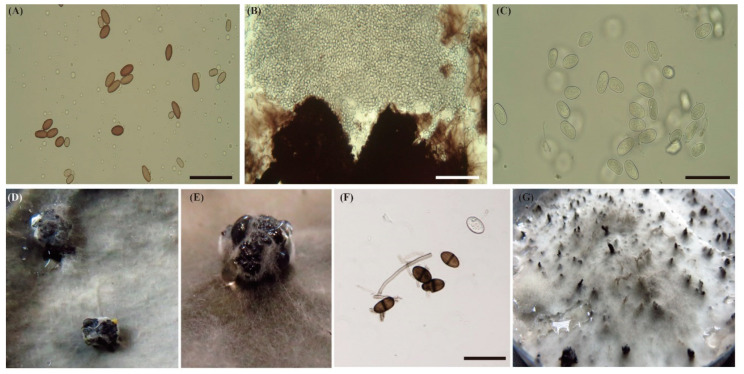
Micrographs of conidial morphology and morphological culture structure on PDA. (**A**) Brown immature conidia of *L. brasiliensis*. Scale bar = 50 µm. (**B**) Pycnidia of *L. hormozganensis* were releasing immature conidia. Scale bar = 50 µm. (**C**) Immature conidia of *L. hormozganensis*. Scale bar = 50 µm. (**D**,**E**) Sporulation of *L. hormozganensis* and *L. pseudotheobromae* on PDA on the four-week colony. (**F**) Mature conidia of *L. theobromae* with dark-brown, one-septate with conspicuous vertical striations. Scale bar = 50 µm. (**G**) The appearance of pycnidia of *L. theobromae* on the surface of PDA with rich sporulation.

From selected woollen mycelium cultures showing similar morphological characteristics of Botryosphaeriaceae, a total of 391 isolates were obtained using the single hyphae method. All isolates were cultured for four weeks to be able to select single spores. The microscopic morphological characteristics of the isolates, such as shape, size, and colour, were observed under the microscope to assist in identifying the species of isolates (Figure 2). Single spores were successfully isolated from a total of 213 samples, including 91 from wax apple (*Syzygium samarangense*), 42 from guava (*Syzygium samarangense*), 43 from mango (*Mangifera indica*), 8 from papaya (*Carica papaya*), 11 from sugar apple (*Annona squamosa*), 9 from cocoa (*Theobroma cacao*), 1 from *Cordia dichotoma*, 2 from banana (*Musa* spp.), 1 from *Alpinia*, and 5 from *Syzygium taiwanicum* (Table 1 and Table S1). To ensure an accurate assessment of the genetic diversities in this study, we only used samples form single spore cultures for subsequent genetic analysis.

Of the 213 isolated single spores, there were 145 and 68 isolates belong to the genus *Lasiodiplodia* and other genus in the family Botryosphaeriaceae, respectively (Table 1 and Table S1). The 145 isolates from the *Lasiodiplodia* species showed typical morphologies of the genus: matured, single septate spore and melanin deposits on the interior surface of the wall arranged longitudinally. These *Lasiodiplodia* isolates were obtained successfully from almost all the host species except *Syzygium taiwanicum*. Out of the 145 *Lasiodiplodia* species, the highest number of isolates were obtained from *L. theobromae* (63 isolates), which accounted for approximately 43.4% and were collected from 19 different locations. The second-highest number of isolates (26 isolates), which accounted for about 17.9%, were obtained from *L. rubropurpurea* and were collected from 10 different locations.

Additionally, the 68 isolates from species of the family Botryosphaeriaceae, including *Neofusicoccum mangiferae*, *N. parvum*, and *Botryosphaeria ramose*, were isolated from wax apple, guava, mango, and *Syzygium taiwanicum*. We included all 213 isolates from the genus *Lasiodiplodia* and other genera in the family Botryosphaeriaceae in the following analyses due to the similarity in colony morphological characteristics and sharing of agricultural hosts between *Lasiodiplodia* and other species (Figure S1). It is worth mentioning that we have identified several novel infection records of *Lasiodiplodia* and *Neofusicoccum* species in the host plants that were not reported before, such as the infection of *L. theobromae* in *Cordia dichotoma* and *Alpinia* (Table 1 and Table S1).

### 3.2. Isolation Frequency (IF) Revealed That L. theobromae Has the Highest IF among Lasiodiplodia Species

**Figure 3:**
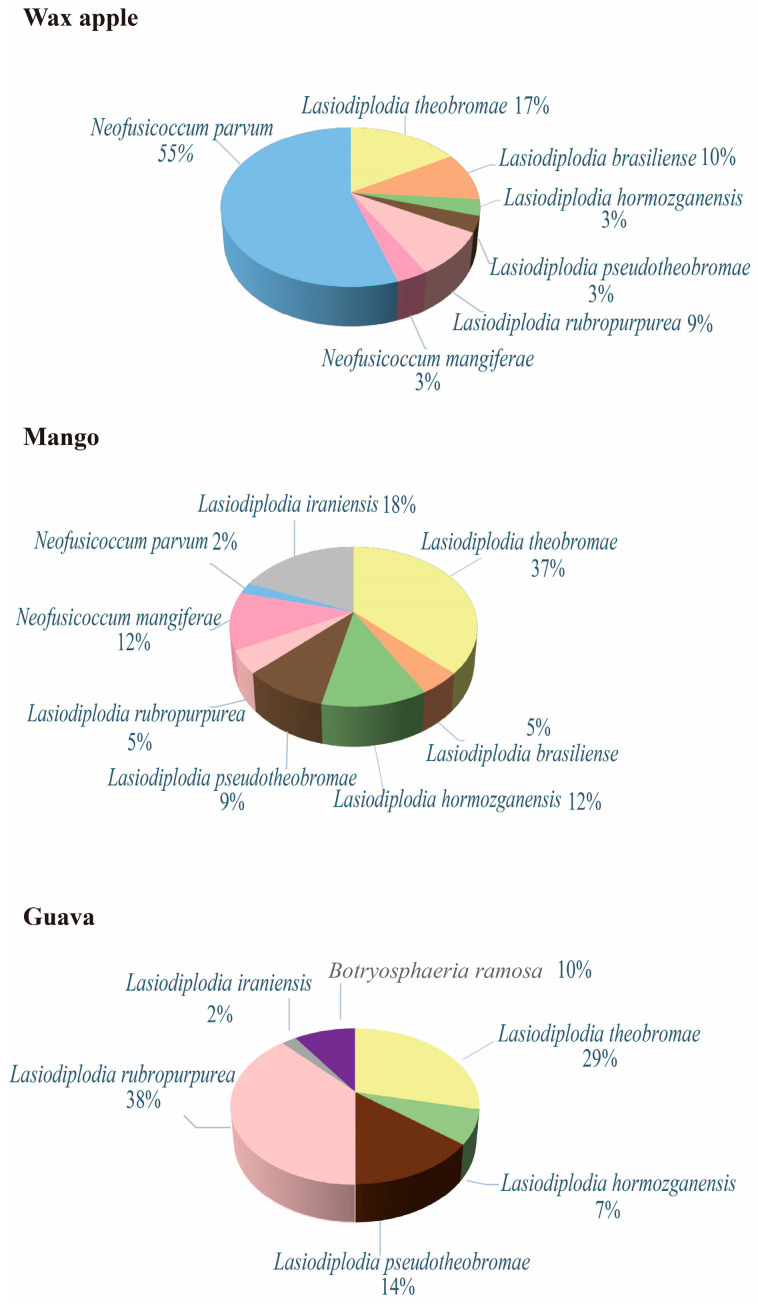
Isolation frequency (IF; %) of fungal species of the family Botryosphaeriaceae isolated from symptomatic fruit plants in Taiwan.

Isolation frequency (IF) was calculated mainly in three host plants, i.e., wax apple, mango and guava, as a sufficient amount of fungal spores were isolated from the three species but not the others (Figure 3). A total of seven fungal species were isolated in wax apples, with the highest percentage of IF of 55% and 17% from *N. parvum* and *L. theobromae*, respectively. A total of eight fungal species were identified in mango, with the highest IF from *L. theobromae* (IF = 37%) and *L. iranensis* (IF = 18%). A total of six fungal species were identified in guava, with the highest IF observed in *L. rubropurpurea* (IF = 38%) and *L. theobromae* (IF = 29%). Based on our findings, it appears that *L. theobromae* has the highest IF among all three fruits in comparison to the *Lasiodiplodia* species (Figure 3 and Table S1).

We further asked whether the percentage of the fungal species was different in each host plant by performing different statistical analyses (Figure S2). The results of ANOVA tests showed that the IF of fungal pathogens in wax apples was significantly different (*p* < 0.01) but not in mango and guava (*p* > 0.05). Tukey tests for differences of means for pathogen isolation frequency further showed that the IF of *N. parvum* was significantly different from other fungal species in wax apples (*p* < 0.001).

### 3.3. Single Molecular Marker Does Not Provide Enough Resolution to Identify Species

Gene genealogies were generated independently for each molecular marker, i.e., ITS, SSU, TEF1, and TUB2, using the Neighbor-Joining method to address the phylogenetic incongruence of *Lasiodiplodia* species. The lengths of each marker are 566 bps of ITS, 1010 bps of SSU, 458 bps of TUB2, and 654 bps of TEF1 (Figures S3–S6). We obtained sequences from GenBank, including 25 ITS from 10 species, 18 SSU from 11 species, 19 TEF1 sequences from 10 species, and 26 TUB2 from 11 species to assist in identifying species at the molecular level.

An overview of the gene genealogies found that only TEF1 sequence fragments could distinguish *L. brasiliensis* from *L. theobromae* and *L. pseudotheobromae* from *L. iranensis* (Figure S5). However, *L. iranensis* could not be separated from the same groupings regardless of which gene genealogy was used. Notably, *L. hormozganensis* was distinguished only in the gene genealogies of TUB2 sequence fragments (Figure S6). Only part of the *L. hormozganensis* samples in the ITS and TEF1 gene genealogy could be separated into a monophyletic group. Upon inspection of the GenBank data (accession number: KP822969.1 and JX464077.1), it was discovered that the ITS sequence data of *L. hormozganensis* published online were also divergent in the Neighbor-Joining tree (Figure S3).

Therefore, we have concluded that using a single molecular marker is insufficient to elucidate the phylogenetic relations of all the species we collected. Using a combined dataset may improve the identification of the phylogenies of the species.

### 3.4. Phylogenetic Analysis Using Combined Molecular Markers Confirmed the Deep Species Identification

**Figure 4:**
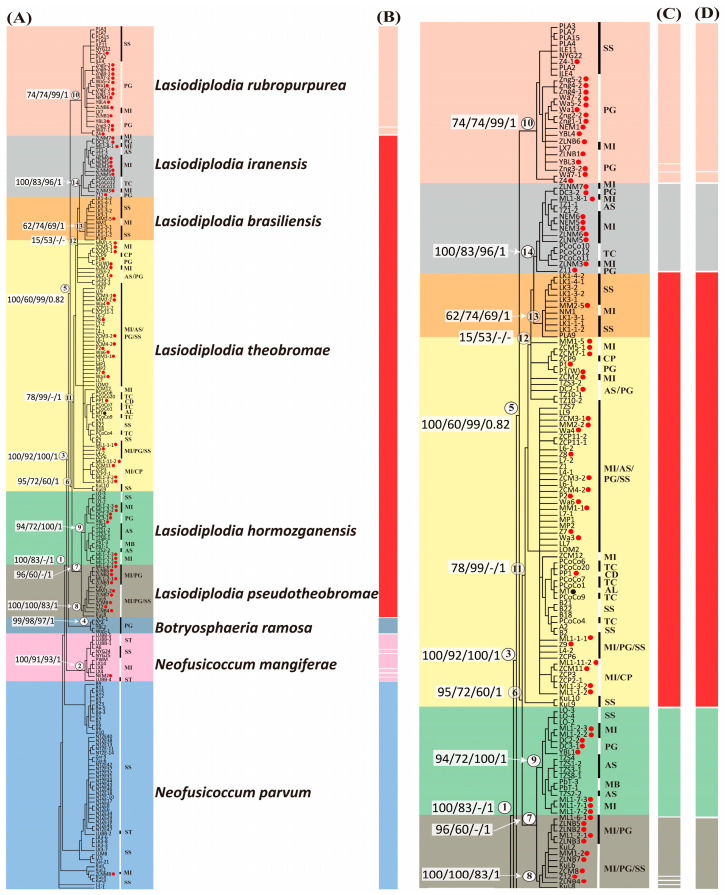
(**A**) The Phylogenetic tree was constructed using Maximum Parsimony of the *Lasiodiplodia* and *Neofusicoccum* species isolated from different fruit species in Taiwan based on DNA sequence data for four loci (SSU, ITS, TEF1, TUB2). Branches were marked with the Maximum Parsimony bootstrap values, Maximum Likelihood bootstrap values, Neighbor-Joining bootstrap values, and Bayesian posterior probabilities, respectively. The red spots indicated that the sample was isolated from the stem. (**B**) ABGD results included six *Lasiodiplodia*, two *Neofusicoccum*, and one *Botryosphaeria* species. (**C**) ABGD results, including six *Lasiodiplodia* species. (**D**) ASAP results included six *Lasiodiplodia* species. [The host species codes] SS: *Syzygium samarangense* (wax apple); PG: *Psidium guajava* (guava); MI: *Mangifera indica* (mango); CP: *Carica papaya* (papaya); AS: *Annona squamosa* (sugar apple); TC: *Theobroma cacao* (cocoa); MB: *Musa basjoo* (banana); CD: *Cordia dichotoma*; AL: *Alpinia*; ST: *Syzygium taiwanicum*. The nodes were labeled with numbers (1–14) encircled by circles.

In order to achieve the purpose of in-depth species identification, we constructed phylogenetic trees using the four-loci dataset of 213 isolates from *Lasiodiplodia*, *Neofusicoccum* and *Botryosphaeria*. Phylogenetic trees using different algorithms, i.e., Maximum Parsimony (MP), Maximum Likelihood (ML), Neighbor-Joining (NJ), and Bayesian inference (BI), found that all produced similar results. A representative phylogenetic tree based on Maximum Parsimony is shown in Figure 4A.

The results showed that using the four-loci dataset provided a much better resolution of species identification than using one locus alone. We confirmed that *N. parvum*, *N. mangiferae*, and *B. ramose* were outgroups of the *Lasiodiplodia* species. All the *Lasiodiplodia* species together formed a monophyletic clade (node 5), which includes the monophyletic clade of *L. rubropurpurea* (node 10) at the basal of the cluster and the monophyletic clade other the *Lasiodiplodia* species (node 6). Under node 6, four monophyletic clades were identified: *L. iranensis* (node 14), *L. brasiliensis* (node 13), *L. hormozganensis* (node 9), and *L. pseudotheobromae* (node 8). All the monophyletic clades mentioned above were supported by at least 60% bootstrap values from MP, ML and NJ methods and over 0.95 probability from Bayesian inference. This means that the identified clades have strong support from the sequence data. It is worth mentioning that *L. rubropurpurea* was identified as a stable and distinct monophyletic group in both gene genealogies using the single marker and the phylogenetic tree using four-loci markers (Figure 4A and Figures S3–S6).

Unlike other species, isolates of *L. theobromae* species were paraphyletic (Figure 4A). Several isolates (11 out of 62) of *L. theobromae* were clustered with *L. iranensis* and *L. brasiliensis* with low support of statistic values (<60% bootstrap value) (node 12). The majority of the isolates of this species (49 out of 62) were further clustered to form a larger clade (node 11). Paraphyletic of the species indicated a possible deep genetic differentiation and diversity within the species. As seen in *L. theobromae*, we observed that *N. parvum* isolates were also paraphyletic, while *N. mangiferae* and *B. ramose* formed two independent monophyletic clades (Figure 4A). Based on these results, we hypothesize that deep genetic differentiation and diversity within *L. theobromae* and *N. parvum* species may exist.

There has been an observation that certain *Lasiodiplodia* species exhibit host-associated clustering of isolates. For instance, isolates from wax apples (host species symbol: SS) of *L. rubropurpurea* were clustered independently from the isolates of the other two hosts, despite being collected from distant locations. Other examples of this phenomenon include *L. iranensis* isolates from cocoa (TC), *L. hormozganensis* isolates from wax apples (SS) and bananas (MB), and *N. mangiferae* isolates from mangoes (MI) (Figure 4A). This highlights the necessity for further analysis of fine-scale genetic clustering.

Taken together, we concluded that utilizing combined four-loci markers provided sufficient resolution in species identification. However, possible deep population structures may exist in *L. theobromae*, requiring further analyses to elucidate the issues fully.

### 3.5. Species Diversity Revealed in Lasiodiplodia Species

We conducted ABGD analysis at two levels (Figure 4B,C and Figure S7). The first level included all species we analyzed (Figure 4B and Figure S7A), while the second level only considered *Lasiodiplodia* species (Figure 4C and Figure S7B). Interestingly, we obtained different results from the two levels, possibly due to gaps between species in different genera. We discovered that most *Lasiodiplodia* species were grouped into one hypothetical species (represented by the red bar) except *L. rubropurpurea* if samples from other genera were included. Within *L. rubropurpurea*, we identified two groups, indicating population divergence (represented by the light orange bars) (Figure 4B). Overall, 11 distinct groups among all species were identified (*p* = 0.001) (Figure 4B and Figure S7A). We further analyzed detailed differentiation within *Lasiodiplodia* species (Figure 4C and Figure S7B). Conversely, more hypothetical species were assigned, resulting in a total of 10 distinct groups being identified within *Lasiodiplodia* species (*p* = 0.001). We observed that *L. iranensis* and *L. hormozganensis* were each assigned as hypothetical species without subgroups in the species. However, multiple groups were identified in *L. rubropurpurea* (three groups) and *L. pseudotheobromae* (four groups). In addition, *L. brasiliensis* and *L. theobromae* were still grouped together.

We further conducted ASAP analysis on the *Lasiodiplodia* clade to confirm the species differentiation identified from ABGD analysis. The results were similar to the ABGD analysis. Other than *L. iranensis* and *L. hormozganensis*, ASAP identified *L. pseudotheobromae* as an independent species without differentiation. However, differentiation was still observed in *L. rubropurpurea* (two groups were identified). Lastly, *L. brasiliensis* and *L. theobromae* were still grouped. Taken together, we concluded that the clade of *L. brasiliensis* is likely not a monophyletic clade as they were suggested to be clustered with *L. theobromae* from both ABGD and ASAP analyses. In addition, a certain degree of differentiation may exist in *L. rubropurpurea* species.

### 3.6. Genetic Diversity and Recombination Events in Lasiodiplodia Species

The genetic diversity data for the four gene regions and the combined dataset were summarized in Table 2. The number of haplotypes (*h*) in the combined dataset ranged from 7 to 27, with the most haplotypes identified in *L. theobromae* (*h* = 27) and the least identified in *L. brasiliensis* (*h* = 7). Haplotype diversity (*Hd*) across all samples ranged from 0.87 to 0.99, with the highest diversity identified in *L. iranensis* (*Hd* = 0.99) using the combined dataset. Nucleotide diversity was found to be highest in *L. rubropurpurea* (*π* = 6.11 × 10^−3^) and lowest in *L. theobromae* (*π* = 1.36 × 10^−3^) using the combined dataset. The highest nucleotide diversity was found in TEF1 loci in *L. rubropurpurea* (*π* = 13.90 × 10^−3^), while the lowest nucleotide diversity was detected in ITS loci in *L. theobromae* (*π* = 0.24 × 10^−3^).

It is worth mentioning that *L. theobromae* has the most haplotypes identified; however, it has the lowest haplotype (*Hd* = 0.87) and nucleotide diversity (*π* = 1.36 × 10^−3^) among all the species using a combined dataset. In particular, ITS in this species showed the lowest diversities for haplotype (*Hd* = 0.12) and nucleotide (*π* = 0.24 × 10^−3^). This indicates a possible population expansion of the species recently. Furthermore, the highest nucleotide diversity of *L. rubropurpurea* may contribute to the multiple groups of the species based on ABGD and ASAP analyses.

We also identified recombination events in *Lasiodiplodia* species. The *R_M_* (minimum number of recombination events, Table 2) values varied between 0 and 2 times for individual genes and between 1 and times for the combined dataset. The combined dataset of *L. theobromae* (*R_M_* = 3) and *L. rubropurpurea* (*R_M_* = 4) showed relatively high numbers of recombination events.

### 3.7. Significant Population Expansion Detected in L. theobromae

We first tested that all four loci were selectively neutral in the genome by examining the degree of linkage disequilibrium (LD), which was evaluated through *Z_nS_* and *ZZ* statistics (Table 2). The significant LD values resulted from fewer recombination events than expected, indicated a possible selection force to link two or more loci together. Our results showed that LD, measured by *Z_nS_*, varied between the lowest value of 0.07 (*L. theobromae*) and the highest of 0.36 (*L. rubropurpurea*). However, the only significant value was observed in *L. brasiliensis* (0.32) using a combined dataset. The *ZZ* values ranged from 0.14 (*L. hormozganensis*) to 0.38 (*L. brasiliensis*) without significant values detected. We then concluded that the loci used in this study were primarily neutral in all the species.

As the four loci were night tightly linked and affected by selection force, we further performed Tajima’s *D* and Fu and Li’s D* tests to assess the neutrality of genomic regions by comparing the observed nucleotide diversity to the expected diversity. These tests assume that all polymorphisms are selectively neutral when the population size remains constant. In other words, the population size change may be reflected by the significant change of test values, indicating the away from the neutrality of the genomic loci. Negative values of the two tests indicate a possible population expansion of the samples. The results of this study indicate that Tajima’s *D* values for each gene fragment ranged from −2.51 to 0.17, while the combined dataset had values ranging from −2.41 to −0.90 (Table 2). Significantly negative Tajima’s *D* values were observed for *Lasiodiplodia* species across various gene fragments and the combined dataset. Fu and Li’s D* values ranged from −4.76 to 1.49 for each gene fragment. When the dataset was combined, the values ranged from −2.66 to −0.16. Based on the combined dataset, both tests provide strong evidence for the population expansion of *L. theobromae*. This is consistent with the species having the highest haplotype numbers and the lowest haplotype and nucleotide diversities observed, as previously mentioned.

Mismatch distribution was used to examine demographic changes by calculating the sum of squared deviations (SSD) and the Harpending’s raggedness index (*H*_Rag_) (Table S2). Based on the spatial expansion model, none of the *Lasiodiplodia* species showed significant values for SSD and *H*_Rag_ (*p* > 0.05). On the other hand, under the demographic expansion model, *H*_Rag_ values were also insignificant (*p* > 0.05). In addition, the mismatch distribution did not indicate a rapid demographic expansion, with multimodal mismatch frequency distribution curves for each *Lasiodiplodia* species (Figure S8).

### 3.8. Genetic Differentiation and Genetic Structure in Lasiodiplodia Species

We conducted genetic differentiation analysis at various levels. First, we employed molecular variance (AMOVA) to classify the overall variations among species, among populations, or within populations (Table 3). The accumulated genetic variation of different classes suggested that the genetic variation primarily accumulated among the *Lasiodiplodia* species (89.18%). The high variability among *Lasiodiplodia* species was significant (*F_CT_* = 0.89). The fixation index among populations within species (*F_SC_*) and the fixation index within populations (*F_ST_*) were significantly higher (*F_SC_* = 0.33; *F_ST_* = 0.93). These results showed that the genetic differentiations are significant at all levels, i.e., among species, among populations and within populations.

Second, pairwise genetic differentiation was assessed by genetic distance values (*F_ST_*) between each of the two *Lasiodiplodia* species (Table S3). *F_ST_* values ranged from 0.26 to 0.87. The lowest value was observed between *L. brasiliensis* and *L. theobromae* (*F_ST_* = 0.26), which was consistent with the results of ABGD and ASAP analyses—the two species were grouped together (Figure 4B–D). The highest *F_ST_* was observed between *L. rubropurpurea* and *L. theobromae* (*F_ST_* = 0.87).

Next, we specifically analyzed pairwise genetic differentiation between host groups within each species (Table S4). Within *L. theobromae*, minimal differentiation was found between guava (PG) and mango (MI) groups (*F_ST_* = 0.01), while the highest differentiation was observed between sugar apple (AS) and wax apple (SS) groups (*F_ST_* = 0.34). The highest genetic differentiation was found in *L. rubropurpurea* between mango (MI) and wax apple (SS) groups (*F_ST_* = 0.92). No differentiation was found among host groups of *L. pseudotheobromae*. *Lasiodiplodia hormozganensis* displayed moderate to high genetic differentiation among most host groups (*F_ST_* = 0.09–0.67), except for the guava and wax apple groups.

**Figure 5:**
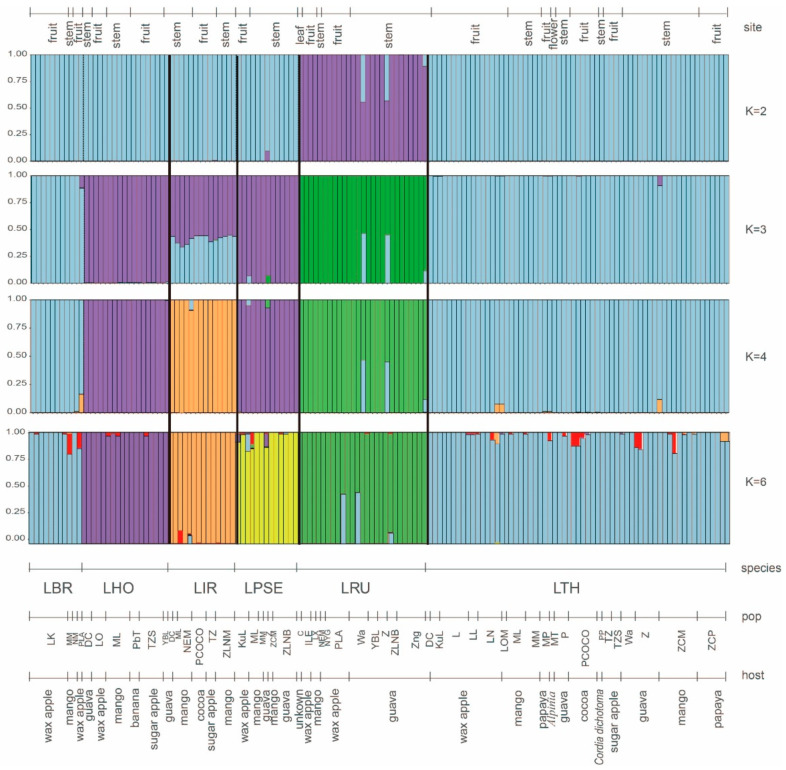
Bar plots for the genetic structure of *Lasiodiplodia* species based on DNA sequence data generated by STRUCTURE. A vertical colour line represents each individual, the same colour indicates that the individual belongs to the same cluster, and black lines separate different species. [Species code] LTH: *L. theobromae*; LBR: *L. brasiliensis*; LHO: *L. hormozganensis*; LPSE: *L. pseudotheobromae*; LRU: *L. rubropurpurea*; LIR: *L. iranensis*. Refer to Table 1 for detailed information on population codes.

Lastly, cluster analysis was performed to investigate the population structure of *Lasiodiplodia* species. The optimal value for K clusters (no. of optimal grouping for the populations) was determined based on the magnitude of ΔK. The best value for K was found to be two, followed by three and four clusters (Figure S9). *Lasiodiplodia rubropurpurea* was clearly distinguished from the other species at K = 2. *Lasiodiplodia pseudotheobromae* and *L. hormozganensis* were grouped together, while *L. brasiliensis* and *L. theobromae* were grouped at K = 4. *Lasiodiplodia iranensis* had a mixed genetic composition at K = 3 but was clearly distinguished at K = 4 (Figure 5). Upon examining K = 6 (analysis result not supported), the grouping situation roughly corresponds to the current species nomenclature, with *L. brasiliensis* and *L. theobromae* being considered the same group, which was consistent with species divergence and genetic differentiation analyses (Figure 4C,D and Table S3).

## 4. Discussion

### 4.1. Genetic Differentiation and Genetic Structure in Lasiodiplodia Species

From the literature, only mango had relatively complete records of Botryosphaeriaceae-related infections in Taiwan and worldwide [26,35,81,82]. The records of infections in other host plants were relatively insufficient in Taiwan. In this study, we reported novel infection records for three species of *Lasiodiplodia* in wax apples, three species in guava and one species each in cocoa and banana (Table 1). These are all important economic fruit plants in Taiwan. Our data also indicated that the isolation frequency varied significantly among host plants. Additionally, the culture morphology of all isolates displayed variability within *Lasiodiplodia* species, and the culture morphology of *Neofusicoccum* species resembled that of *Lasiodiplodia* species. Plants affected by Botryosphaeriaceae species displayed similar disease symptoms but with varying levels of aggressiveness [6,20,24,25,26,27]. This increases the complexity of disease management. Notably, our study revealed genetic differences in pathogens among different infected crops. It is essential to consider these genetic differences when developing disease management strategies.

The intraspecific variation in ITS sequences of *Lasiodiplodia* was generally low, as described by Alves et al. (2008) [83]. Conversely, TEF1 sequences exhibited relatively high intraspecific variation. This makes it a suitable marker for identifying different *Lasiodiplodia* species except for *L. hormozganensis*, which could only be clearly identified in the TUB2 gene segment. Different *Lasiodiplodia* species showed varying levels of nucleotide diversity across different sequence data. *L. rubropurpurea* had the highest diversity in TEF1 and four-gene fragment data, *L. iranensis* in TUB2 and SSU, and *L. brasiliensis* in ITS. Surprisingly, the most widespread *L. theobromae* did not display the highest diversity in any of the examined sequence data. The haplotype diversity and nucleotide diversity values of *L. theobromae* were similar to previous studies [84]. The high genetic diversity of *Lasiodiplodia* species in Taiwan may indicate a strong evolutionary potential and long-standing possibilities. Furthermore, the high haplotype and nucleotide diversity can be attributed to a secondary contact between differentiated lineages or a lack of ancient population bottleneck or founder effect. Similarly, high genetic diversity can be attributed to a large population maintained over evolutionary time due to habitat stability [85,86].

### 4.2. Phylogenetic Relationships and Species Delimitation within the Lasiodiplodia Genus

Phylogenetic reconstructions revealed that *L. iranensis*, *L. brasiliensis*, and *L. theobromae* had the closest phylogenetic relationship. Further analyses using ABGD, ASAP, and STRUCTURE showed that *L. brasiliensis* and *L. theobromae* were more closely related. The close phylogenetic relationship between *L. brasiliensis* and *L. theobromae* agrees with other phylogenetic studies [26,87,88,89,90,91,92,93,94,95]. The analysis of MP, ML, and BI trees showed that *L. pseudotheobroma* had the closest relationship with *L. hormozganensis*, while *L. iranensis* was closely related to *L. brasiliensis* and *L. theobromae*. This pattern was consistent with some previous phylogenetic studies [18,26,89,91,92,96] but contrary to other studies [30,93,95,97].

It is speculated that the discrepancies in phylogenetic relationship patterns may be due to the differences in the number and types of *Lasiodiplodia* species included in phylogenetic analyses of different studies. This indicates that the lack of comprehensive sampling of genetic diversity can affect the construction of phylogenetic relationships for the genus *Lasiodiplodia*. Insufficient sampling and too few gene analyses may cause discrepancies in phylogenetic relationship patterns. Therefore, it is essential to conduct extensive sampling of *Lasiodiplodia* species and use different genetic markers to examine phylogeny, genetic diversity, and genetic structure, which can help to solve the uncertain taxonomic status of this complex group. Furthermore, given the close relationship among *Lasiodiplodia* species, incomplete lineage sorting and introgression may pose problems, resulting in false signals and turbulence in species discrimination and phylogenetic relationship patterns. This speculation is supported by the inconsistent results of the gene trees based on different gene fragments in our study. The individual gene genealogies and combined dataset displayed inconsistent phylogenetic patterns, especially the relationship among *L. pseudotheobromae*, *L. hormozganensis*, and *L. iranensis* species. These observed incongruences confirm that the loci might involve distinct evolutionary histories [98]. The inconsistency of the gene genealogies was a signal that indicated the existence of incomplete lineage sorting and introgression. Other previous studies also mentioned the inconsistency of the gene genealogies of the *Lasiodiplodia* species [30,89]. The construction of the phylogenetic relationships of the most recently diverged species is challenging, as incomplete lineage sorting predominates at shallow time depths. The difficulties arising from incomplete lineage sorting have been extensively described [99,100,101,102]. The instability in constructing phylogenetic relationships also highlights the importance of using other genetic markers to clarify the relationship of each *Lasiodiplodia* species.

DNA sequence analysis is essential for defining species boundaries in the genus *Lasiodiplodia*. However, discrepancies between phylogenetic loci have made it challenging to delimit species accurately. Applying various species delimitation methods and consistently estimating species diversity can increase credibility, and inconsistent results imply varying delineation powers of multiple methods. In the case of inconsistent findings, it is recommended that species should be designated conservatively so that they can genuinely represent evolutionary metapopulation lineages [103].

This study showed that *L. rubropurpurea* harboured more than one distinct group with high support based on ABGD and ASAP analyses, proving that *L. rubropurpurea* might be a multiple-species complex. In the phylogenetic analysis of the combined four-loci dataset, *L. brasiliensis* was found to be a separate group from *L. theobromae*, but with low support. ABGD and ASAP analyses and structure-based clustering did not provide evidence for *L. brasiliensis* as a stable species. The original identification report for *L. brasiliensis* also lacked strong support for its classification, with overlapping morphological characteristics with *L. theobromae* [104,105]. The high-haplotype and low-nucleotide diversity in *L. brasiliensis* and *L. theobromae* demonstrate that this group should be recognized as a distinct lineage rather than a single species. Previous studies identified *L. brasiliensis* as a distinct species based on concatenating a few genes [104] without examining incongruences between gene genealogies. This study suggests that *L. brasiliensis* is mainly attributed to the intraspecific variability in *L. theobromae*, causing the clade to be incorrectly recognized as a unique lineage.

### 4.3. Signs of Population Expansion, Subdivision, and Cryptic Sex in Lasiodiplodia Species

*Lasiodiplodia theobromae* exhibited significant negative Tajima’s *D* and Fu and Li’s D* values, which may indicate either positive selection (selective sweeps) or a population expansion. The presence of recombination suggested that this species complex might have recently expanded by producing many offspring. Furthermore, the mismatch distribution analysis showed a multimodal mismatch frequency distribution with nonsignificant values in all the *Lasiodiplodia* species, suggesting the population has experienced a recent demographic expansion that followed a decrease in population size due to the subdivisions of each *Lasiodiplodia* species. This subdivision was also supported by the presence of host-associated differentiation in other genetic analyses.

The sexual structure of *Lasiodiplodia* species has rarely been observed in nature, and only a few species have had their sexual stages described [8,10,11,12,13]. These species are generally considered to reproduce primarily through asexual reproduction mainly. However, cryptic sex evidence could be inferred from the phylogenetic tree pattern, proving that some species had long been considered asexual but underwent cryptic sex [106,107,108]. For sexual fungi, the phylogenetic tree of combined multigene sequence data was expected to be poorly resolved compared with the respective gene tree since each gene has a different evolutionary history and presents different topologies due to the recombination of sexual fungi. However, a branch with high support in the combined phylogenetic tree indicated genetic isolation [74]. Most ascomycetes have both sexual and asexual reproductive systems, and wholly cloned fungi were rare. Even in species whose sexual stages were unknown, molecular markers could often reveal a certain degree of recombination [106,107,109]. This study analyzed the minimum number of recombination events (*R_M_*) and showed that *Lasiodiplodia* species presented cryptic recombination events, even though the values were not high, indicating the possibility of cryptic sex reproduction.

### 4.4. Intraspecies Variations and Host-Associated Genetic Differentiation

The study found that multiple isolates of the *L. theobromae* species were separated into diverse groups with low bootstrap values. The fixation index also suggested that the *Lasiodiplodia* species presented significant genetic differentiation within the species level despite the low genetic variation. The *F_CT_* was significantly higher than *F_SC_*, indicating a strong population genetic structure at the group scale [110]. This aspect might imply that the present cryptic species or genetic structure in *L. theobromae* has not yet been discovered due to a few samples used in previous studies. Other studies have also presented a similar state that the *L. theobromae* clade could not form a single group with low posterior probability support among internal branches [6,90]. It indicated a considerable intraspecific diversity and might hide several cryptic species in *L. theobromae* [18,19,22,83,111,112,113,114].

Fungi often had descriptions of cryptic species or host races. With the development of multilocus phylogenetic analysis, many cryptic species of *Lasiodiplodia* have been discovered and defined [18,22,111,114,115]. This study’s phylogenetic trees and genetic differentiation analysis illustrated that *L. theobromae* and other *Lasiodiplodia* species revealed intraspecies variations related to the host differences. Different host species had a robust disruptive selection, and these systems continue to respond in coevolutionary dynamics. The disruptive selection caused by host variation can drive the parasite population to differentiate, even in symbiotics, and yield new host races or sibling species. This situation is easy to find in fungi that are primarily asexual [116,117]. Genetic differentiation and specialists on one or a few related hosts of generalist fungal pathogen have been reported in the Botryosphaeriaceae [25,32]. This study demonstrated that the *L. theobromae* complex is an ongoing evolutionary lineage and had recently diverged into unique clades, such as *L. brasiliensis*, and genetic groups presented a host-association genetic differentiation pattern. The findings of this study validated the inferences of Slippers et al. (2013) and De Wet et al. (2008) studies.

## 5. Conclusions

This study examined the genetic diversity and cryptic species structure of *Lasiodiplodia* fungi that infect fruit trees in Taiwan. The results showed various species of *Lasiodiplodia* present in fruit crops, often coinfecting with other Botryosphaeriaceae species. The phylogenetic diversity of *Lasiodiplodia* in Taiwan was underestimated, and the inconsistent phylogeny of *Lasiodiplodia* species indicated incomplete lineage sorting and introgression. The study also identified *L. rubropurpurea* as a multiple-species complex and did not support that *L. brasiliensis* was an excellent species delineation. The study suggested that the species identification in *Lasiodiplodia* had been largely overestimated due to the lack of integrated and accurate species delineation analysis for such a genus with complex evolutionary patterns and high intraspecific variability.

Host-associated differentiation was also observed, with some *Lasiodiplodia* species exhibiting high specificity towards certain host plants. The study detected evidence of recent sudden population growth and cryptic sex reproduction in *Lasiodiplodia* species. Although *Lasiodiplodia* species, especially *L. theobromae*, caused sporadic fruit tree diseases in Taiwan, and pesticides usually had inhibitory effects, this study denoted that some *Lasiodiplodia* species had a high evolutionary capacity. Therefore, future pesticide control experiments should take note of this. Overall, this study provides new insights into the genetic diversity and taxonomy of *Lasiodiplodia* fungi infecting fruit trees in Taiwan. It highlights the importance of using multiple gene regions for species delimitation and that can aid the development of *Lasiodiplodia* species management strategies in Taiwan that emphasize the importance of monitoring different hosts of *Lasiodiplodia* species.

## Figures and Tables

**Table 1:** Lists of *Lasiodiplodia* species isolated in Taiwan. Hosts and numbers of isolates were listed for each species. Bold text indicates new host records. Common name for each species is as follows: *Syzygium samarangense* (wax apple), *Psidium guajava* (guava), *Mangifera indica* (mango), *Carica papaya* (papaya), *Annona squamosa* (sugar apple), *Theobroma cacao* (cocoa), and *Musa* spp. (banana). The detailed locations, isolates and population codes are listed in Table S1.

Taxon	Host	No. of Isolates (No. of Locations)
*Lasiodiplodia theobromae*	*Syzygium samarangense* (wax apple)	15 (4)
	*Psidium guajava* (guava)	12 (4)
	*Mangifera indica* (mango)	16 (4)
	*Carica papaya* (papaya)	8 (2)
	*Annona squamosa* (sugar apple)	4 (2)
	*Theobroma cacao* (cocoa)	6 (1)
	***Cordia dichotoma***	1 (1)
	***Alpinia***	1 (1)
*Lasiodiplodia brasiliensis*	***Syzygium samarangense* (wax apple)**	9 (2)
	*Mangifera indica* (mango)	2 (2)
*Lasiodiplodia hormozganensis*	***Syzygium samarangense* (wax apple)**	3 (1)
	*Mangifera indica* (mango)	5 (1)
	***Psidium guajava* (guava)**	3 (2)
	*Annona squamosa* (sugar apple)	5 (1)
	***Musa* spp. (banana)**	2 (1)
*Lasiodiplodia pseudotheobromae*	*Syzygium samarangense* (wax apple)	3 (1)
	*Psidium guajava* (guava)	6 (2)
	*Mangifera indicnga* (mango)	4 (3)
*Lasiodiplodia rubropurpurea*	***Syzygium samarangense*** (wax apple)	8 (3)
	***Psidium guajava*** (guava)	16 (5)
	*Mangifera indica* (mango)	2 (2)
*Lasiodiplodia iranensis*	*Mangifera indica* (mango)	8 (3)
	***Psidium guajava*** (guava)	1 (1)
	*Annona squamosa* (sugar apple)	2 (1)
	***Theobroma cacao* (cocoa)**	3 (1)
**Total**		**145**

**Table 2:** An estimate of genetic variability and neutrality indices of *Lasiodiplodia* species. *S*: The number of segregating sites, *h*: The number of haplotypes, *Hd*: The haplotype diversity, π: Nucleotide diversity. *R_M_*: the minimum number of recombination events, *Z_nS_*: Kelly’s *Z_Ns_* [62], *ZZ*: Rozas’ *ZZ* [65]. Statistical significance: * *p* < 0.05; ** *p* < 0.01.

	*S*	*h*	*Hd*	*π* (×10^−3^)	*Z_nS_*	*ZZ*	*R_M_*	Fu and Li’s *D* *	Tajima’s *D*
*L. theobromae*									
ITS	2	3	0.12	0.24	0.00	0.00	0	0.72	−1.19
SSU	18	11	0.29	0.66	0.11	0.22	0	−4.76 *	−2.51 **
EF1	31	12	0.70	3.99	0.17	0.13	2	−1.22	−2.08 *
TUB	6	6	0.24	0.70	0.07	0.13	0	−0.63	−1.84 *
Combined dataset	57	27	0.87	1.36	0.07	0.24	3	−2.66 *	−2.41 **
*L. brasiliensis*									
ITS	6	6	0.80	3.73	0.54	0.12	1	−0.44	−0.23
SSU	11	3	0.35	1.98	0.82	0.08	0	−2.43 *	−2.01 *
EF1	6	2	0.18	1.85	1.00	0.00	0	−2.21 *	−1.85 *
TUB	2	2	0.18	0.81	1.00	0.00	0	−1.66	−1.43
Combined dataset	25	7	0.87	2.11	0.32 **	0.38	1	−2.12 *	−1.70
*L. hormozganensis*									
ITS	6	6	0.86	3.46	0.13	−0.06	0	1.26	0.09
SSU	6	6	0.49	2.30	0.15	0.14	0	−3.03 *	−2.15 **
EF1	2	2	0.11	0.38	1.00	0.00	0	−1.99	−1.51
TUB	6	5	0.66	3.06	0.17	0.15	0	−0.10	−0.70
Combined dataset	22	13	0.95	1.68	0.10	0.14	1	−1.23	−1.30
*L. pseudotheobromae*									
ITS	2	2	0.15	0.60	ND	ND	0	−1.78	−1.47
SSU	15	7	0.73	3.12	0.31	0.05	0	−1.37	−1.45
EF1	15	7	0.73	3.12	0.31	0.05	0	−1.37	−1.45
TUB	3	2	0.15	1.03	1.00	0.00	0	−2.02	−1.65
Combined dataset	26	10	0.95	2.04	0.19	0.21	1	−1.91	−1.65
*L. rubropurpurea*									
ITS	1	2	0.14	0.28	ND	ND	0	0.61	−0.73
SSU	13	6	0.40	1.21	0.48	0.22	1	−3.20 *	−2.16 *
EF1	44	8	0.84	13.90	0.61	0.18	1	1.49 *	−1.12
TUB	2	3	0.15	0.33	0.00 **	0.00	0	−2.22	−1.51
Combined dataset	60	16	0.90	6.11	0.36	0.35	4	0.16	−1.48
*L. iranensis*									
ITS	−	1	−	−	−	−	−	−	−
SSU	13	6	0.40	1.21	0.48	0.22	1	−3.20 *	−2.16 *
EF1	18	5	0.67	9.59	0.37	−0.04	0	−0.99	−0.61
TUB	8	9	0.91	6.58	0.22	−0.05	1	0.02	0.17
Combined dataset	37	13	0.99	3.72	0.18	0.20	2	−1.29	−0.90

**Table 3:** Summary of molecular variance (AMOVA) of *Lasiodiplodia* species on the sequence data. *, *p* < 0.05.

	Sum of Squares	*d.f.*	Variance Components	Percentage of Variation	Fixation Indices
Among species	2629.35	5	24.02	89.18	*F_CT_* = 0.89 *
Among populations within species	207.57	46	0.97	3.60	*F_SC_* = 0.33 *
Within populations	182.76	94	1.94	7.22	*F_ST_* = 0.93 *

## Data Availability

The GenBank accession numbers are as follow: OR534007-OR534219 for ITS region; OR552184–OR552396 for TEF1 region, OR534310-OR534522 for SSU region and OR551773–OR551985 for TUB2.

## Supplementary Materials

The following supporting information can be downloaded at: https://www.mdpi.com/article/10.3390/jof9090950/s1, Table S1: Lists of isolate number, location, host and population code of *Lasiodiplodia*, *Neofusicoccum* and *Botryosphaeria* species. Bold text indicates new host records; Table S2: Estimated demographic parameters using the mismatch distribution analysis of *Lasiodiplodia* species; Table S3: List of pairwise genetic distance values (*F_ST_*) among *Lasiodiplodia* species based on sequence data; Table S4: List of pairwise genetic distance values (*F_ST_*) values among different host species of *Lasiodiplodia* species based on sequence data. The grey area represents *F_ST_* values among different host species within the same species. Note: [Species code] LTH: *L. theobromae*; LBR: *L. brasiliensis*; LHO: *L. hormozganensis*; LPSE: *L. pseudotheobromae*; LRU: *L. rubropurpurea*; LIR: *L. iranensis*. [Host species code] SS: *Syzygium samarangense* (wax apple); PG: *Psidium guajava* (guava); MI: *Mangifera indica* (mango); CP: *Carica papaya* (papaya); AS: *Annona squamosa* (sugar apple); TC: *Theobroma cacao* (cocoa); MB: *Musa basjoo* (banana); Figure S1: *Lasiodiplodia* and *Neofusicoccum*, species colony morphology, grew on PDA after four weeks at 25 °C; Figure S2: The average isolation frequencies (%) belonging to the Botryosphaeriaceae family in wax apple, guava, and mango hosts. The data presented show the mean ± standard error. The significance level was determined using one-way ANOVA and Tukey simultaneous tests to compare the means. *p* < 0.01 indicates statistical significance. Species codes: LTH: *L. theobromae*; LBR: *L. brasiliensis*; LHO: *L. hormozganensis*; LPSE: *L. pseudotheobromae*; LRU: *L. rubropurpurea*; LIR: *L. iranensis*; NM: *N. mangiferae*; NP: *Neofusicoccum parvum*; BR: *Botryosphaeria ramose*; Figure S3: The phylogenetic relationships of the *Lasiodiplodia* and *Neofusicoccum* species isolated from fruit plants in Taiwan were constructed based on ITS sequence data and integration with the NCBI database. Bootstrap values by the Neighbor-Joining method were above branches; Figure S4: The phylogenetic relationships of the *Lasiodiplodia* and *Neofusicoccum* species isolated from fruit plants in Taiwan were constructed based on SSU sequence data and integration with the NCBI database. Bootstrap values by the Neighbor-Joining method were above branches; Figure S5: The phylogenetic relationships of the *Lasiodiplodia* and *Neofusicoccum* species isolated from fruit plants in Taiwan were constructed based on TEF1 sequence data and integration with the NCBI database. Bootstrap values by the Neighbor-Joining method were above branches; Figure S6: The phylogenetic relationships of the *Lasiodiplodia* and *Neofusicoccum* species isolated from fruit plants in Taiwan were constructed based on TUB2 sequence data and integration with the NCBI database. Bootstrap values by the Neighbor-Joining method were above branches; Figure S7: Automatic Barcode Gap Discovery (ABGD) results presented the number of partitions obtained in each prior threshold for (A), including six *Lasiodiplodia*, two *Neofusicoccum*, and one *Botryosphaeria* species. (B) included six *Lasiodiplodia* species; Figure S8: Mismatch distribution analysis in *Lasiodiplodia* species using sequence data from combined four-loci. The X-axis displays the observed and expected pairwise differences in nucleotides, while the Y-axis displays their frequencies; Figure S9: The number of groups K was detected using the delta K values and mean LnP(K) (mean log-likelihood values) by STRUCTURE for six *Lasiodiplodia* species based on DNA sequence data.
