# Supplementary material for: Unveiling Cryptic Species Diversity and Genetic Variation of Lasiodiplodia (Botryosphaeriaceae, Botryosphaeriales) Infecting Fruit Crops in Taiwan
Source: J Fungi (Basel). 2023 Sep 20;9(9):950. doi: 10.3390/jof9090950 (PMC10532828; doi:10.3390/jof9090950)
Supplement: Supplementary file 1 [file jof-09-00950-s001.zip › jof-2600531-supplementary.pdf]

Supplementary Materials

Table S1: Lists of isolate number, location, host and population code of *Lasiodiplodia*, *Neofusicoccum* and *Botryosphaeria* species. Bold text indicates new host records.

Taxon	Host	Location	Population code	No. of isolates
Lasiodiplodia theobromae	*Syzygium samarangense* (wax apple)	Linbian Township, Pingtung County	LTH (L)	6
		Linbian Township, Pingtung County	LTH (LL)	2
		Linbian Township, Pingtung County	LTH (LN)	5
		Changjhih Township, Pingtung County	LTH (KuL)	2
	Psidium guajava (guava)	Yanchao Dist., Kaohsiung City	LTH (Z)	5
		Yanchao Dist., Kaohsiung City	LTH (Wa)	3
		Dajia Dist., Taichung City	LTH (DC)	1
		Danei Dist., Tainan City	LTH (P)	3
	Mangifera indica (mango)	Maolin Dist., Kaohsiung City	LTH (ML)	4
		Nansi Dist., Tainan City	LTH (ZCM)	8
		Taoyuan Dist., Kaohsiung City	LTH (MM)	3
		Linluo Township, Pingtung County	LTH (LOM)	1
	Carica papaya (papaya)	Nansi Dist., Tainan City	LTH (ZCP)	6
		Kaohsiung City	LTH (MP)	2
	Annona squamosa (sugar apple)	Taimali Township, Taitung County	LHO (TZS)	2
		Taimali Township, Taitung County	LHO (TZ)	2
	Theobroma cacao (cocoa)	Neipu Township, Pingtung County	LHO (PCOCO)	6
	Cordia dichotoma	Danei Dist., Tainan City	LTH (PP)	1
	Alpinia	Yanchao Dist., Kaohsiung City	LTH (MT)	1
Lasiodiplodia brasiliensis	***Syzygium samarangense* (wax apple)**	Liouguei Dist., Kaohsiung City	LBR (LK)	8
		Neipu Township, Pingtung County	LBR (PLA)	1
	Mangifera indica (mango)	Taoyuan Dist., Kaohsiung City	LBR (MM)	1
		Kaohsiung City	LBR (NM)	1
Lasiodiplodia hormozganensis	***Syzygium samarangense* (wax apple)**	Linluo Township, Pingtung County	LHO (LO)	3
	Mangifera indica (mango)	Maolin Dist., Kaohsiung City	LHO (ML)	5
	***Psidium guajava* (guava)**	Dajia Dist., Taichung City	LHO (DC)	2
		Yanchao Dist., Kaohsiung City	LHO (YBL)	1
	Annona squamosa (sugar apple)	Taimali Township, Taitung County	LHO (TZS)	5
	***Musa* spp. (banana)**	Taimali Township, Taitung County	LHO (PbT)	2
Lasiodiplodia pseudotheobromae	*Syzygium samarangense* (wax apple)	Changjhih Township, Pingtung County	LPSE (KuL)	3
	Psidium guajava (guava)	Yanchao Dist., Kaohsiung City	LPSE (Z)	1
		Tianliao Dist., Kaohsiung City	LPSE (ZLNB)	5

	*Mangifera indic*nga (mango)	Maolin Dist., Kaohsiung City	LPSE (ML)	2
		Taoyuan Dist., Kaohsiung City	LPSE (MM)	1
		Nansi Dist., Tainan City	LPSE (ZCM)	1
Lasiodiplodia rubropurpurea	***Syzygium samarangense*** (wax apple)	Yanchao Dist., Kaohsiung City	LRU (NYG)	1
		Neipu Township, Pingtung County	LRU (PLA)	5
		Yuanshan Township, Yilan County	LRU (ILE)	2
	Psidium guajava (guava)	Yanchao Dist., Kaohsiung City	LRU (Z)	2
		Yanchao Dist., Kaohsiung City	LRU (Wa)	4
		Yanchao Dist., Kaohsiung City	LRU (Zng)	6
		Yanchao Dist., Kaohsiung City	LRU (YBL)	2
		Tianliao Dist., Kaohsiung City	LRU (ZLNB)	2
	Mangifera indica (mango)	Lucao Township, Chiayi County	LRU (LX)	1
		Sinying Dist., Tainan City	LRU (NEM)	1
Lasiodiplodia iranensis	*Mangifera indica* (mango)	Maolin Dist., Kaohsiung City	LIR (ML)	1
		Sinying Dist., Tainan City	LIR (NEM)	3
		Tianliao Dist., Kaohsiung City	LIR (ZLNM)	4
	Psidium guajava (guava)	Dajia Dist., Taichung City	LIR (DC)	1
	Annona squamosa (sugar apple)	Taimali Township, Taitung County	LIR (TZ)	2
	***Theobroma cacao* (cocoa)**	Neipu Township, Pingtung County	LIR (PCOCO)	3
Neofusicoccum mangiferae	***Syzygium samarangense*** (wax apple)	Yanchao Dist., Kaohsiung City	NEOM (NYG)	2
		Linbian Township, Pingtung County	NEOM (LN)	1
	Mangifera indica (mango)	Lucao Township, Chiayi County	NEOM (LX)	3
		Sinying Dist., Tainan City	NEOM (NEM)	1
		Neipu Township, Pingtung County	NEOM (PWM)	1
	Syzygium taiwanicum	Lanyu Township, Taitung County	NEOM (LUBB)	4
Botryosphaeria ramosa	***Psidium guajava*** (guava)	Yanchao Dist., Kaohsiung City	BOTR (Wa)	1
		Yanchao Dist., Kaohsiung City	BOTR (YBL)	1
		Nansi Dist., Tainan City	BOTR (ZC)	2
Neofusicoccum parvum	***Syzygium samarangense*** (wax apple)	Linbian Township, Pingtung County	NEOP (L)	2
		Linbian Township, Pingtung County	NEOP (LL)	2
		Liouguei Dist., Kaohsiung City	NEOP (LK)	4

	Meishan Township, Chiayi County	NEOP (Zai)	3
	Changjhih Township, Pingtung County	NEOP (KuL)	4
	Linbian Township, Pingtung County	NEOP (LN)	13
	Xinyi Township, Nantou County	NEOP (NTZE)	22
Syzygium taiwanicum	Lanyu Township, Taitung County	NEOP (LUBB)	1
Mangifera indica (mango)	Tianliao Dist., Kaohsiung City	NEOP (ZLNM)	1
Total			213

Table S2: Estimated demographic parameters using the mismatch distribution analysis of *Lasiodiplodia* species.

Taxon	Locus	Spatial expansion		Demographic expansion	
		SSD	H_{Rag}	SSD	H_{Rag}
L. theobromae	ITS	0.00	0.59	0.00	0.59
	SSU	0.01	0.28	0.01	0.28
	TEF1	0.00	0.03	0.31*	0.03
	TUB2	0.00	0.37	0.00	0.37
	Combined	0.00	0.02	0.00	0.02
L. brasiliensis	ITS	0.03	0.10	0.03	0.10
	SSU	0.03	0.32	0.04	0.32
	TEF1	0.02	0.74	0.05*	0.75
	TUB2	0.02	0.74	0.06	0.74
	Combined	0.05	0.09	0.05	0.09
L. hormozganensis	ITS	0.02	0.09	0.02	0.09
	SSU	0.00	0.10	0.32*	0.10
	TEF1	0.01	0.81	0.12	0.81
	TUB2	0.03	0.11	0.03	0.11
	Combined	0.01	0.05	0.01	0.05
L. pseudotheobromae	ITS	0.01	0.76	0.03*	0.76
	SSU	0.02	0.05	0.62*	0.05
	TEF1	0.02	0.11	0.02	0.11
	TUB2	0.03	0.04	0.03	0.04
	Combined	0.01	0.03	0.21*	0.03
L. rubropurpurea	ITS	0.00	0.53	0.00	0.53
	SSU	0.00	0.20	0.01	0.20
	TEF1	0.03	0.04	0.03	0.04
	TUB2	0.00	0.53	0.00	0.53
	Combined	0.01	0.02	0.01	0.02
L. iranensis	ITS	na	na	na	na
	SSU	0.00	0.11	0.44*	0.11
	TEF1	0.04	0.22	0.11	0.22
	TUB2	0.02	0.05	0.01	0.05
	Combined	0.01	0.02	0.14*	0.02

Table S3: List of pairwise genetic distance values (F_{ST}) among *Lasiodiplodia* species based on sequence data.

	L. theobromae	*L. brasiliensis*	*L. hormozganensis*	*L. pseudotheobromae*	*L. rubropurpurea*	*L. iranensis*
L. theobromae						
L. brasiliensis	0.26					
L. hormozganensis	0.80	0.78				
L. pseudotheobromae	0.85	0.82	0.69			
L. rubropurpurea	0.87	0.85	0.86	0.86		
L. iranensis	0.75	0.71	0.80	0.77	0.82	

Table S4: List of pairwise genetic distance values (F_{ST}) values among different host species of *Lasiodiplodia* species based on sequence data. The grey area represents F_{ST} values among different host species within the same species. Note: [Species code] LTH: *L. theobromae*; LBR: *L. brasiliensis*; LHO: *L. hormozganensis*; LPSE: *L. pseudotheobromae*; LRU: *L. rubropurpurea*; LIR: *L. iranensis*. [Host species code] SS: *Syzygium samarangense* (wax apple); PG: *Psidium guajava* (guava); MI: *Mangifera indica* (mango); CP: *Carica papaya* (papaya); AS: *Annona squamosa* (sugar apple); TC: *Theobroma cacao* (cocoa); MB: *Musa basjoo* (banana).

		LTH (SS)	LTH (PG)	LTH (MI)	LTH (CP)	LTH (AS)	LBR (SS)	LHO (SS)	LHO (MI)	LHO (PG)	LHO (AS)	LPSE (SS)	LPSE (PG)	LRU (SS)	LRU (PG)	LIR (MI)	LIR (AS)
LTH	(PG)	0.05															
LTH	(MI)	0.07	0.01														
LTH	(CP)	0.11	0.08	0.06													
LTH	(AS)	0.34	0.15	0.15	0.21												
LTH	(TC)	0.17	0.07	0.08	0.13	0.06											
LBR	(MI)						0.05										
LHO	(MI)							0.24									
LHO	(PG)							0.00	0.09								
LHO	(AS)							0.25	0.20	0.11							
LHO	(MB)							0.67	0.16	0.15	0.33						
LPSE	(PG)											-0.02					
LPSE	(MI)											0.00	0.03				
LRU	(PG)													0.41			
LRU	(MI)													0.92	0.08		
LIR	(AS)															0.29	
LIR	(TC)															0.29	0.75

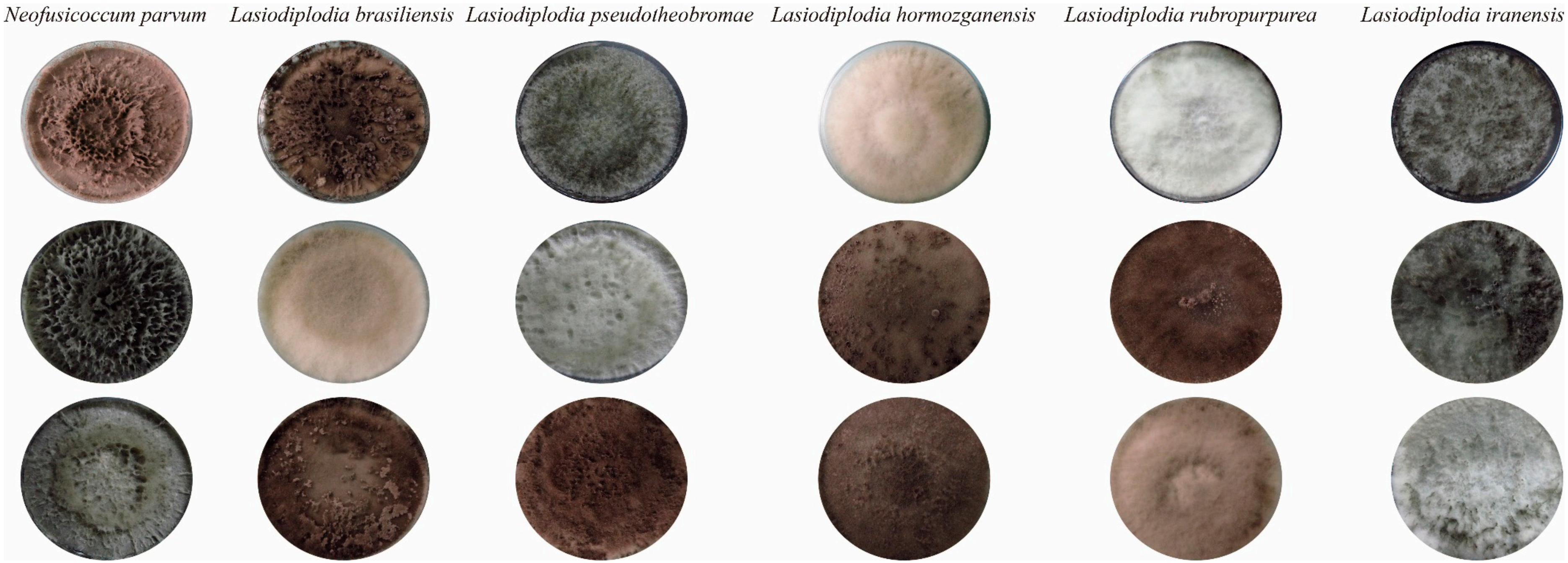

Figure S1: *Lasiodiplodia* and *Neofusicoccum*, species colony morphology, grew on PDA after four weeks at 25 °C.

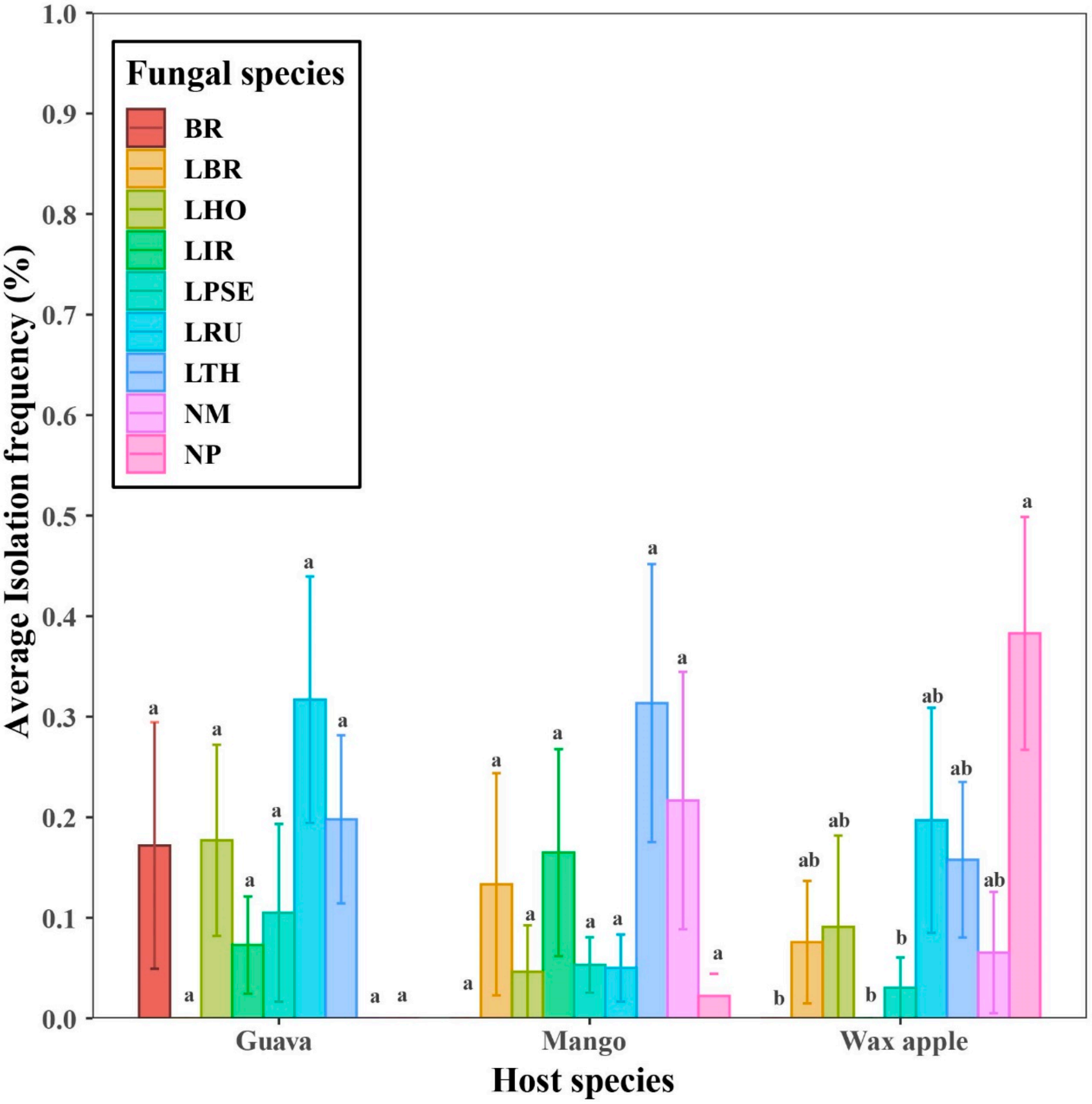

Figure S2: The average isolation frequencies (%) belonging to the Botryosphaeriaceae family in wax apple, guava, and mango hosts. The data presented show the mean ± standard error. The significance level was determined using one-way ANOVA and Tukey simultaneous tests to compare the means. $p < 0.01$ indicates statistical significance. Species codes: LTH: *L. theobromae*; LBR: *L. brasiliensis*; LHO: *L. hormozganensis*; LPSE: *L. pseudotheobromae*; LRU: *L. rubropurpurea*; LIR: *L. iranensis*; NM: *N. mangiferae*; NP: *Neofusicoccum parvum*; BR: *Botryosphaeria ramosa*.

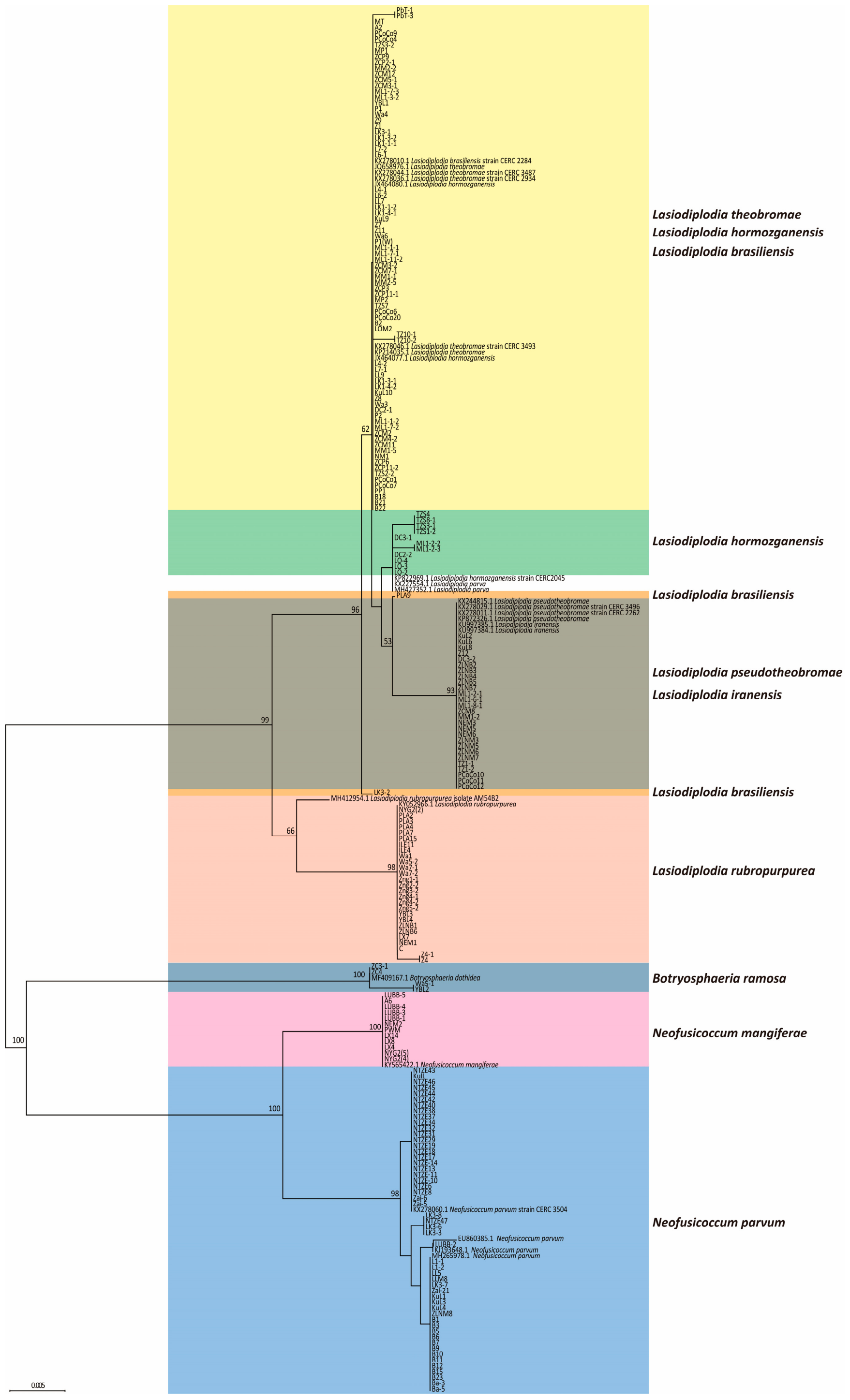

Figure S3: The phylogenetic relationships of the *Lasiodiplodia* and *Neofusicoccum* species isolated from fruit plants in Taiwan were constructed based on ITS sequence data and integration with the NCBI database. Bootstrap values by the Neighbor-Joining method were above branches.

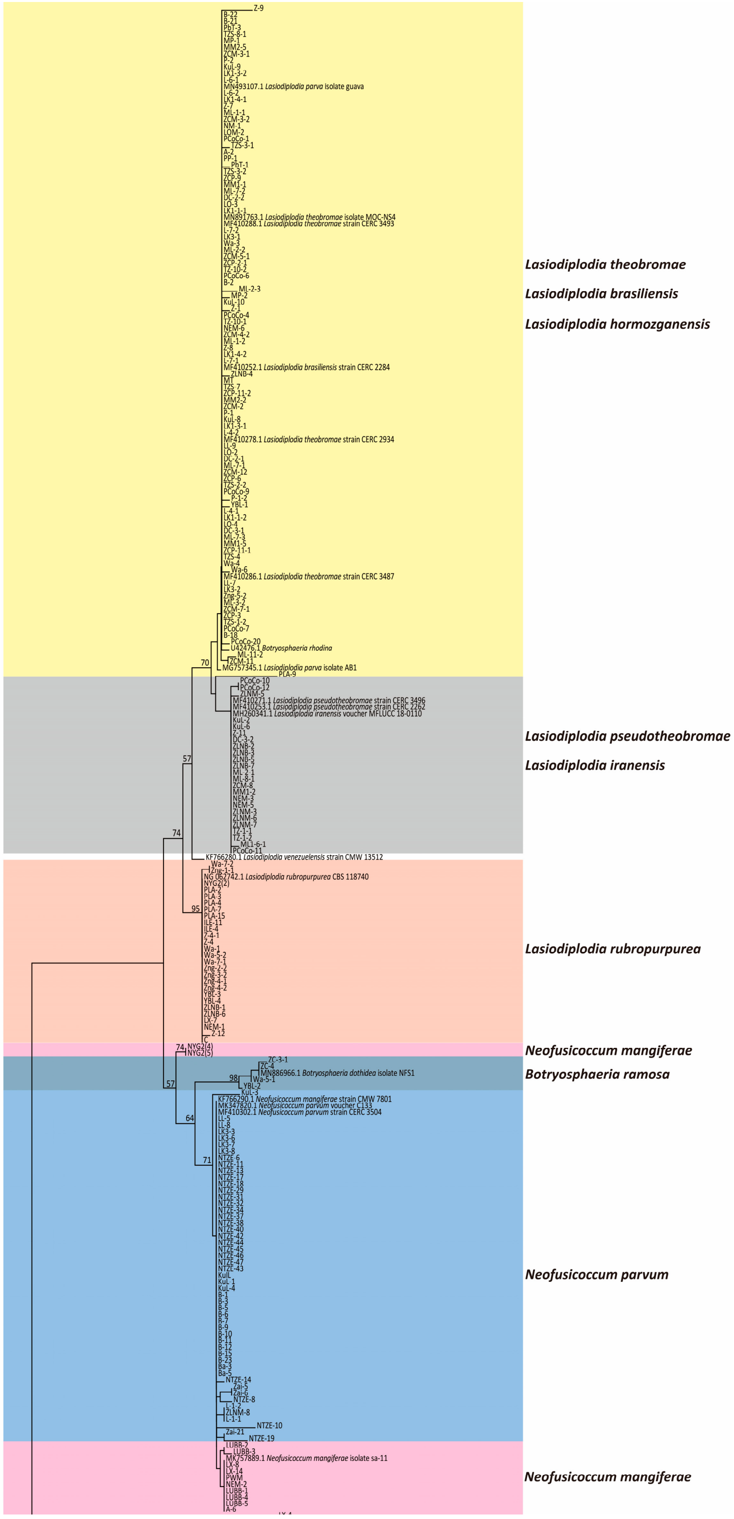

Figure S4: The phylogenetic relationships of the *Lasiodiplodia* and *Neofusicoccum* species isolated from fruit plants in Taiwan were constructed based on SSU sequence data and integration with the NCBI database. Bootstrap values by the Neighbor-Joining method were above branches.

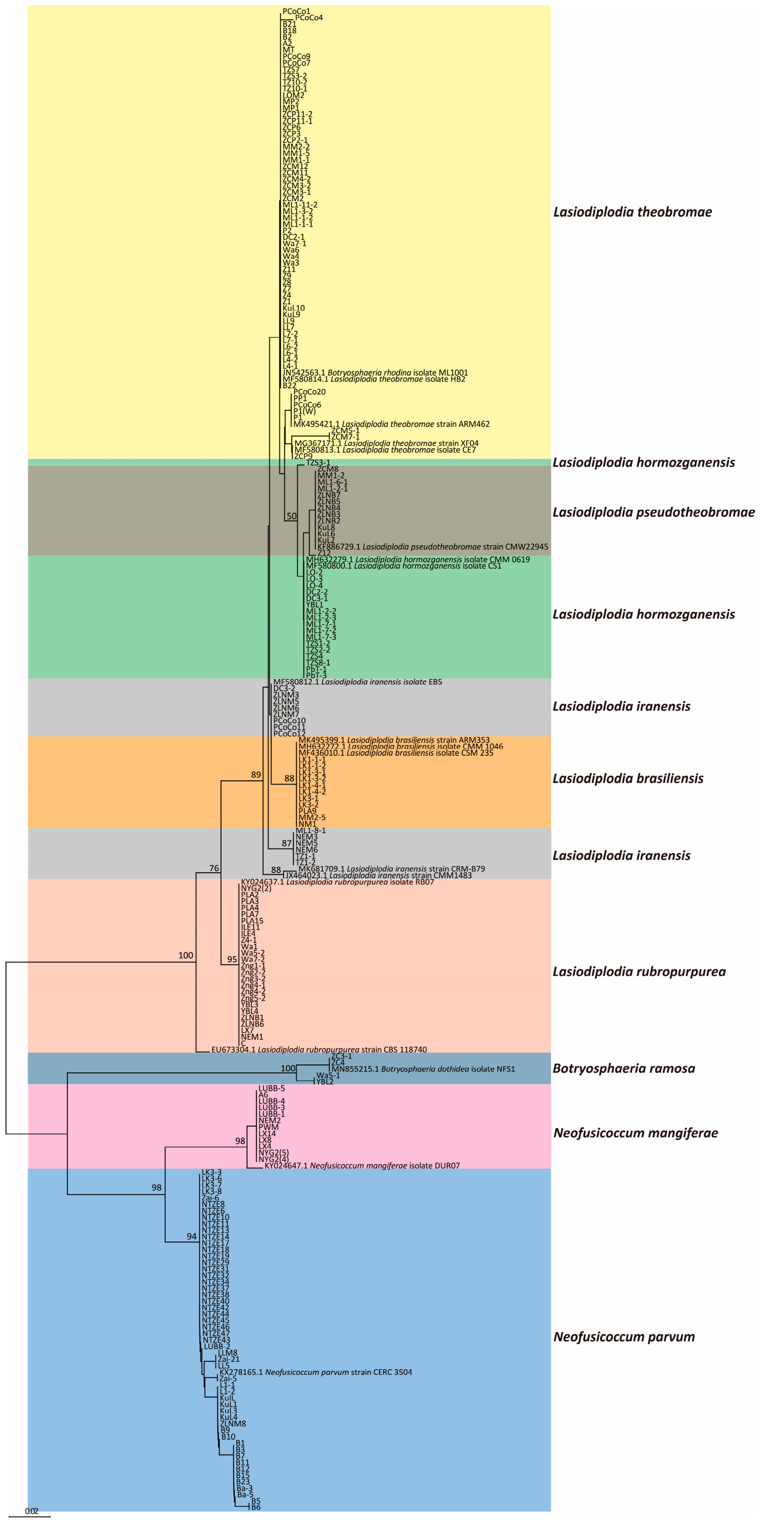

Figure S5: The phylogenetic relationships of the *Lasiodiplodia* and *Neofusicoccum* species isolated from fruit plants in Taiwan were constructed based on TEF1 sequence data and integration with the NCBI database. Bootstrap values by the Neighbor-Joining method were above branches.

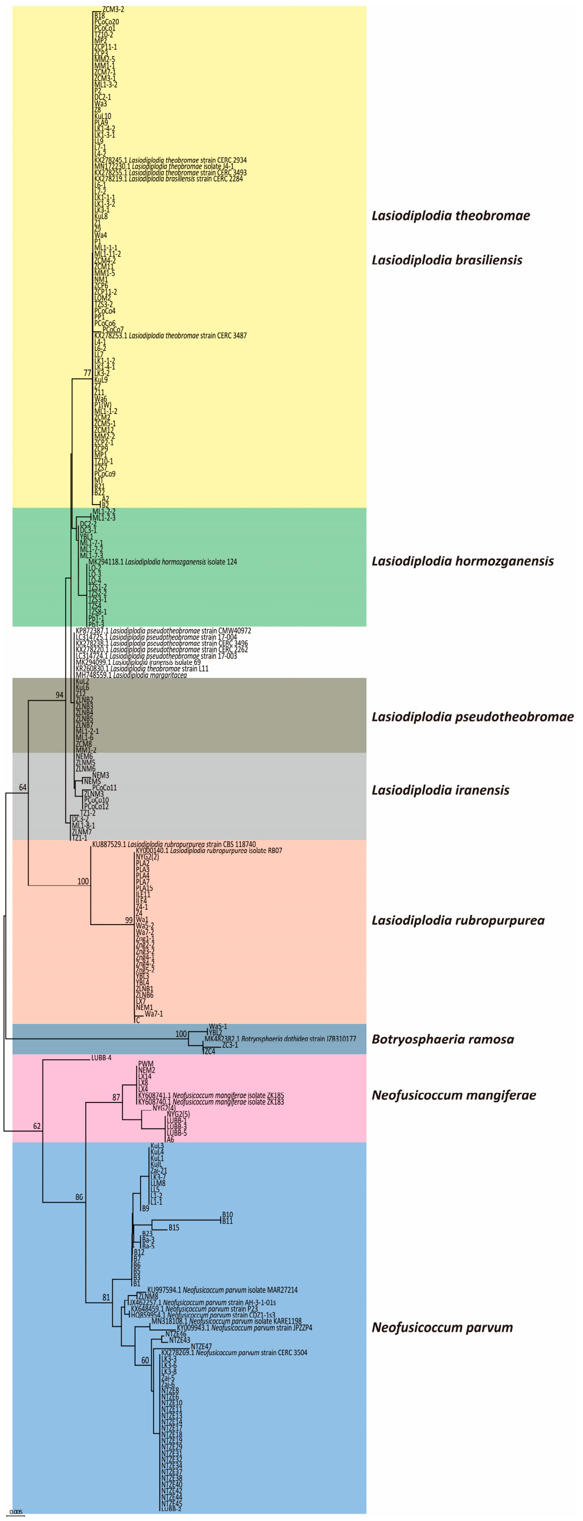

Figure S6: The phylogenetic relationships of the *Lasiodiplodia* and *Neofusicoccum* species isolated from fruit plants in Taiwan were constructed based on TUB2 sequence data and integration with the NCBI database. Bootstrap values by the Neighbor-Joining method were above branches.

(A)

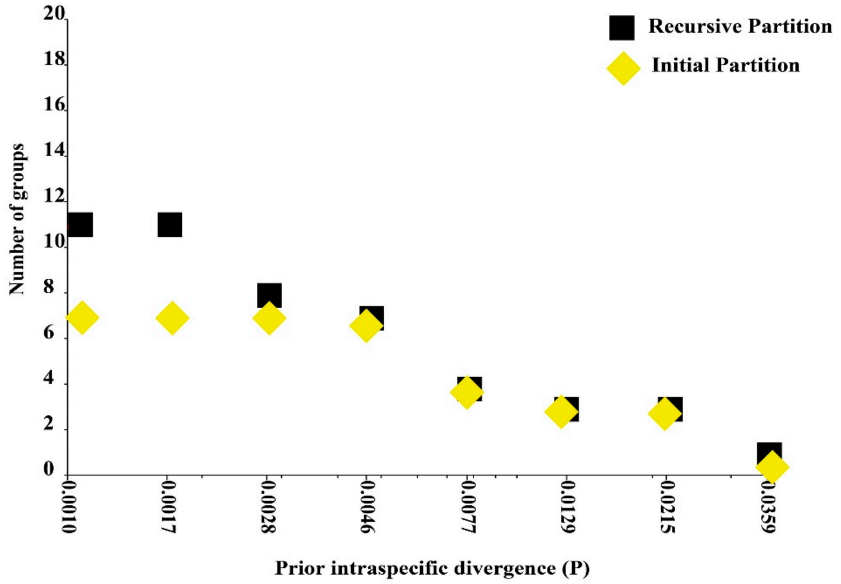

(B)

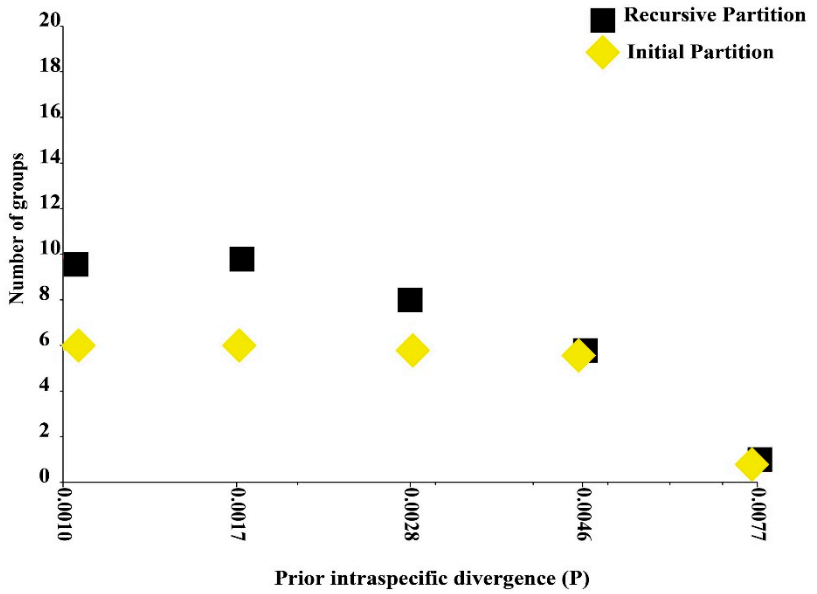

Figure S7: Automatic Barcode Gap Discovery (ABGD) results presented the number of partitions obtained in each prior threshold for (A), including six *Lasiodiplodia*, two *Neofusicoccum*, and one *Botryosphaeria* species. (B) included six *Lasiodiplodia* species.

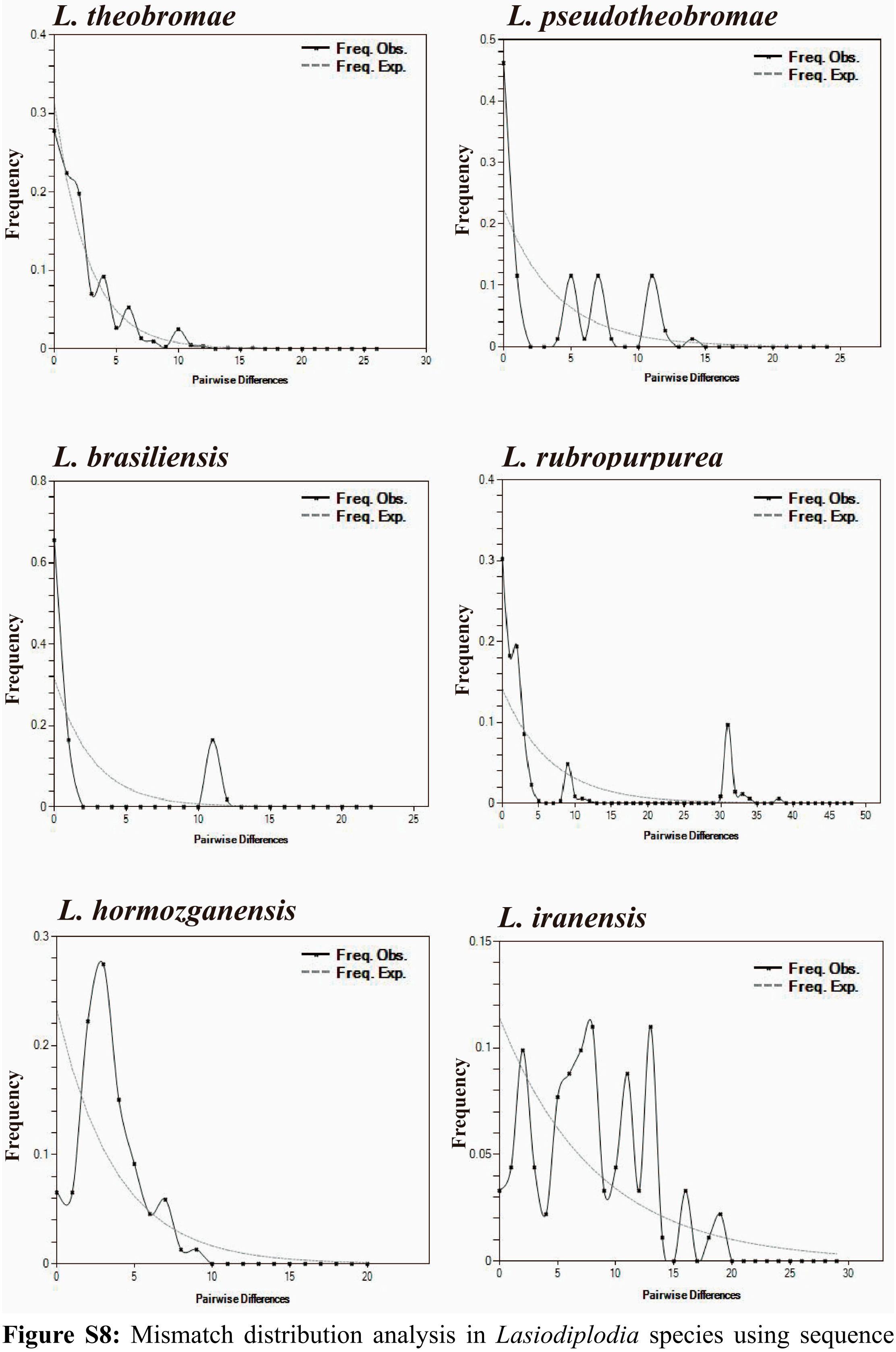

Figure S8: Mismatch distribution analysis in *Lasiodiplodia* species using sequence data from combined 4-loci. The X-axis displays the observed and expected pairwise differences in nucleotides, while the Y-axis displays their frequencies.

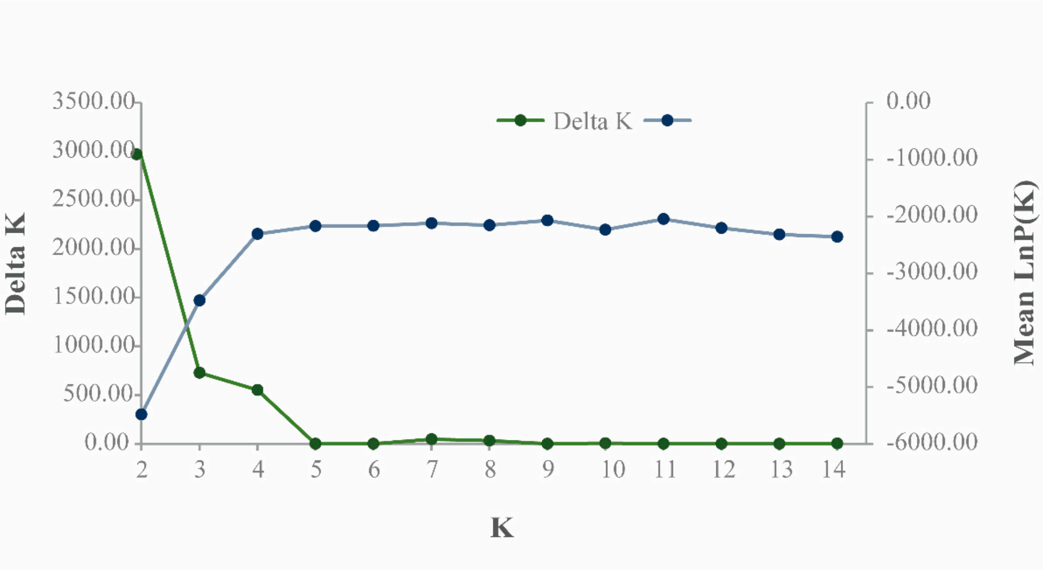

Figure S9: The number of groups K was detected using the delta K values and mean LnP(K) (mean log-likelihood values) by STRUCTURE for six *Lasiodiplodia* species based on DNA sequence data.
